# Network structure and fluctuation data improve inference of metabolic interaction strengths with the inverse Jacobian

**DOI:** 10.1038/s41540-024-00457-y

**Published:** 2024-11-23

**Authors:** Jiahang Li, Wolfram Weckwerth, Steffen Waldherr

**Affiliations:** 1 Molecular Systems Biology Lab (MOSYS), Department of Functional and Evolutionary Ecology, University of Vienna, Vienna, Austria; 2 School of Mathematical Sciences, Nankai University, Tianjin, China; 3 Vienna Molecular Metabolomics Center (VIME), University of Vienna, Vienna, Austria

**Keywords:** Computational biology and bioinformatics, Biochemical networks, Systems analysis

## Abstract

Based on high-throughput metabolomics data, the recently introduced inverse differential Jacobian algorithm can infer regulatory factors and molecular causality within metabolic networks close to steady-state. However, these studies assumed perturbations acting independently on each metabolite, corresponding to metabolic system fluctuations. In contrast, emerging evidence puts forward internal network fluctuations, particularly from gene expression fluctuations, leading to correlated perturbations on metabolites. Here, we propose a novel approach that exploits these correlations to quantify relevant metabolic interactions. By integrating enzyme-related fluctuations in the construction of an appropriate fluctuation matrix, we are able to exploit the underlying reaction network structure for the inverse Jacobian algorithm. We applied this approach to a model-based artificial dataset for validation, and to an experimental breast cancer dataset with two different cell lines. By highlighting metabolic interactions with significantly changed interaction strengths, the inverse Jacobian approach identified critical dynamic regulation points which are confirming previous breast cancer studies.

## Introduction

With the advancement of next-generation sequencing and single-cell technology^1^, we can now generate a plethora of OMICS measurements within a short time frame. A primary focus of systems biology is to analyze such data to determine molecular interactions and the mechanisms through which these interactions influence the function and behavior of the system^2,3^. One key goal in metabolomics is to understand how changes in interactions between or kinetics of enzymes and metabolites induce or reflect phenotypic differences^4–9^. From a systematic perspective, metabolic networks typically involve many non-linear relationships among metabolites and enzymes, where perturbations on each compound play a crucial role^10–12^. While covariance analysis of the measurements can reveal some relevant connections between the involved compounds, it cannot uncover the system’s interaction kinetics and dynamic regulation. The aim of this work is to find interactions and regulation points with significantly changed interaction strength between two different conditions, by combining the covariance matrix obtained from measurements with additional information on network structure and fluctuation sources.

Current systematic approaches for metabolomics data analysis can be divided into three categories: statistical analysis, kinetic modeling, and network analysis. Statistical methods, particularly machine learning algorithms provide valuable insights into cellular activities under various treatment conditions and identify key biomarkers within the biological system^13,14^. These methods encompass techniques such as Principal Components Analysis (PCA)^15^, clustering analysis^16^, deep learning^17^, genetic algorithm^18^ and boosting machine learning methods^19,20^. However, they lack the ability to detect perturbation sites associated with the dynamics of the underlying metabolic network system. On this aspect, kinetic models can be constructed to improve our systemic insight into a metabolic network. Over the last two decades, extensive biological studies have developed manually curated or optimized kinetic models which are available in databases such as the BioModels Database^21^.

To analyze dynamic regulations of the system, it is natural to build kinetic models fitting the metabolomics measurements. During this modeling process, kinetic parameters can be obtained from previous models^22^ or estimated from experimental data. Overall, the modeling process is a long and iterative endeavor involving literature mining and parameter tuning, and sometimes it does not yield satisfactory results^23,24^. Moreover, constructing a comprehensive biological model often necessitates time-series data, which is not always available due to experimental constraints.

Another mathematical approach uses network analysis on the system’s Jacobian matrix, often beginning with the examination of a correlation or covariance matrix estimated from the measurements^9,10,25^. Subsequent studies aim to uncover underlying causal relationships from the correlation network^12,26^. The available comprehensive information about metabolic network structure is now being collected in different databases^27–29^, and can be used as extra information for the interaction network inference^30,31^. This approach to network inference relies on stochastic fluctuations, which induce variability in metabolite concentrations that can be used to infer metabolic interactions even in steady state with an inverse Jacobian algorithm^25^. In recent years, several studies have focused on the steady-state Jacobian analysis of metabolomics data^32–36^. The Jacobian matrix represents the dynamic mechanism of the metabolic network and is highly related to the control coefficients in the metabolic control theory^37,38^. Jacobian analysis can thus guide the metabolic dynamics manipulation^39,40^ and it is critical to understand the interaction strengths and kinetics of the system. The differential Jacobian reflects the changes in dynamic regulation between two phenotypes (e.g. health and disease) and can be further utilized to uncover new metabolic mechanisms^41–43^. Steuer^32^ developed a structural modeling approach to investigate the steady-state probability encompassing all possible explicit kinetic parameters. Jamshidi et al. developed a kinetic approach with mass action modeling using fluxomics and metabolomics data to calculate the system steady-state Jacobian^33,34^. Using time-series metabolomics data, Nägele^36^ investigated the Metabolic regulation of subcellular sucrose cleavage with Hessian matrices; Akbari et al., developed a dynamic mode analysis approach to decompose the reaction networks into different physically important timescales, which tightly connect to Jacobian eigenvalues^35^. In addition, several studies have developed inverse differential Jacobian algorithms using only metabolomics measurements with many samples, which provide a convenient way to infer differences in the dynamics of metabolic networks between different conditions^6,30,31,41–45^.

**Fig. 1:**
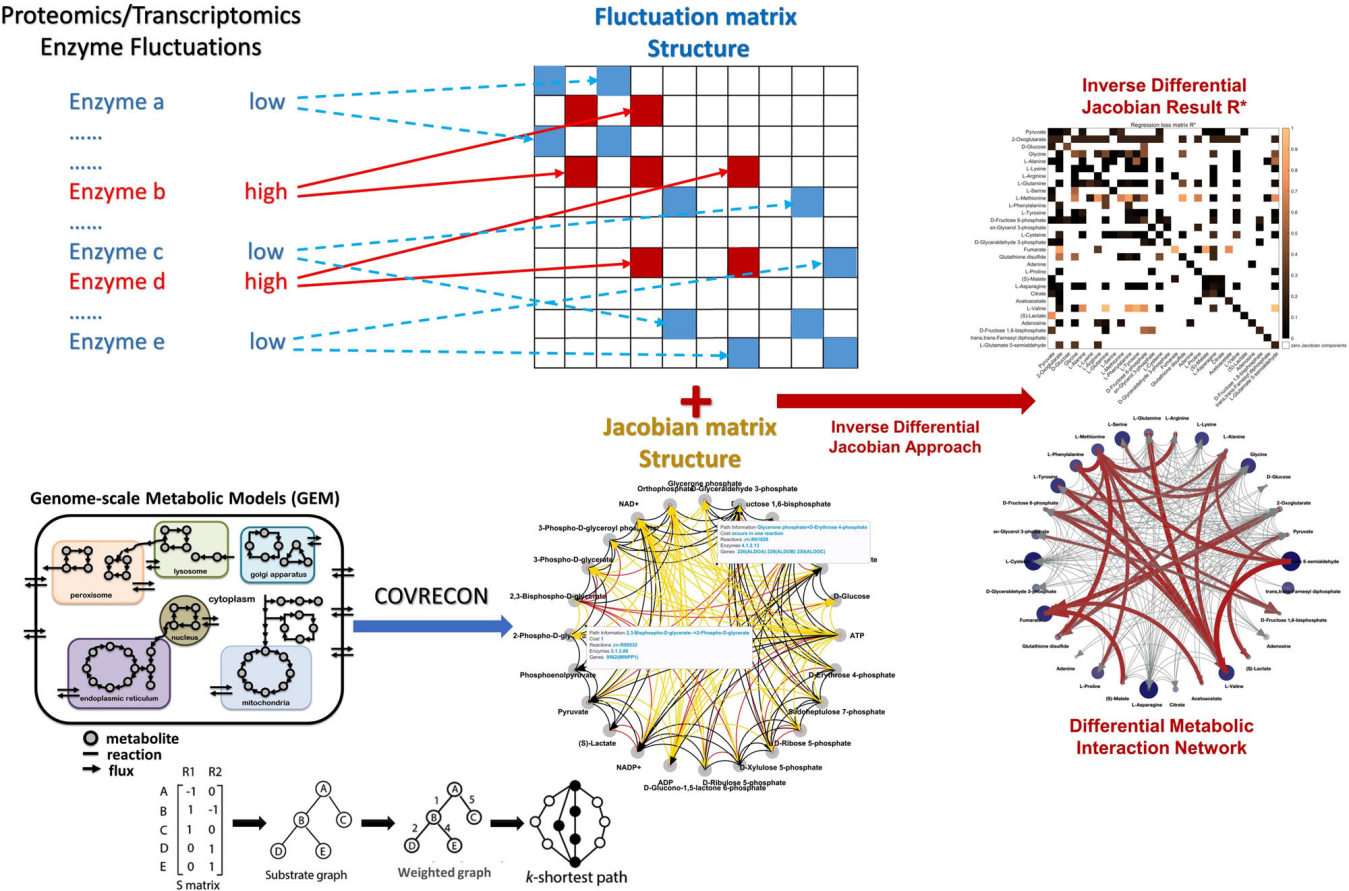
Work scheme. the inverse Jacobian analysis with a non-diagonal fluctuation matrix (adapted from^30^, CC-BY). This approach involves three steps: step 1, construct the metabolic interaction network and generate corresponding Jacobian matrix structure; step 2, construct the fluctuation matrix D structure as described in Method section; step 3, use other omics datasets (transcriptomics or proteomics) to set constraints on D; step 4, do the inverse Jacobian analysis, apply D structure and D constraints (if available in step 3) during the sampling step in the inverse Jacobian algorithm. Eventually, we are able to calculate a regression loss matrix R* to represent the differential Jacobian matrix DJ and the metabolic interaction regulations between two conditions.

The inverse Jacobian approaches assume that the variations of steady-state metabolomics measurements in one condition originate from fluctuations in the systems^12,25,30^. These fluctuations reflect the stochastic properties of biochemical reactions within the cell. In these previous studies, the fluctuations are claimed to independently act on all metabolites^25,30,31,44,46^. Such fluctuations would primarily arise from stochastically perturbed transport between the cells and their external environments. However, more recent studies have provided evidence that fluctuations also originate from within the network itself^47–53^. Over the last years, it has been shown that fluctuations in the gene regulatory network result in variations in enzyme activities, either due to variations in enzyme expression or in post-translational regulation, further leading to variations in the reaction rate parameters^50,52,53^. Due to the reaction network structure, this leads to correlated fluctuations acting on multiple metabolites. More specifically, we define a fluctuation matrix D to be used in the inverse Jacobian algorithm, which in former algorithms was assumed diagonal, while the fluctuations we consider here give rise to a non-diagonal structure. Moreover, in contrast to previous approaches, we reconstruct the fluctuation matrix D from the network structure, using constraints based on the variance of the enzyme activities. Figure 1 presents a scheme of this approach. By utilizing the reaction network information in the structure of both the Jacobian J and the fluctuation matrix D, we can improve the regression-loss based inverse differential Jacobian algorithm. Using this inverse Jacobian approach, we calculate a regression loss matrix R* to represent the differential Jacobian matrix DJ. Large values in the regression loss matrix reflect critical changes in metabolic interactions strength between the two phenotypes.

We evaluate the proposed approach with several published models collected from the EBI BioModels database^21^, and also apply the method to a breast cancer dataset with two different conditions^54^. We compared the inverse Jacobian results with different assumptions on the structure of the fluctuation matrix D. We analyzed the precision and accuracy of our algorithm. This analysis is based on varying three factors: (1) the amplitude of fluctuations, (2) the number of off-diagonal components in the fluctuation matrix D, and (3) the fluctuation magnitude of off-diagonal components compared to the diagonal components in the fluctuation matrix D. The main findings are that the inverse differential Jacobian algorithm is significantly enhanced using the non-diagonal structure with an integrative sampling; nevertheless, it remains feasible to assume a diagonal structure of D in the inverse differential Jacobian algorithm when the number of off-diagonal fluctuations is large or their magnitude is relatively small. In conclusion, this article gives the first comprehensive analysis of the impact of non-diagonal fluctuations on the inverse Jacobian approach and largely enhances the original algorithm by deducing the fluctuation structure. Furthermore, using the enzyme activity data as fluctuation constraints, we introduce an integrative sampling approach, which further enhances the inverse Jacobian algorithm. This approach holds significant promise for improving our understanding of metabolic network dynamics and the robustness of the inverse Jacobian algorithm in various applications.

## Results

### Improved differential Jacobian reconstruction when considering off-diagonal fluctuations

As shown in the methods section, if noise acts on reaction parameters, the fluctuation matrix D has a non-diagonal structure. In Eq. (6) given in the Methods section, we derived the resulting structure of D as a sum of two matrices accounting for the fluctuations acting on compounds directly (diagonal) and on reaction parameters (both off-diagonal and diagonal), respectively. While the relevant structure information can be determined from just the reaction stoichiometry, determining the relative magnitudes of the off-diagonal elements requires enzyme activity data. In this section, we compare the results of the inverse Jacobian algorithm taking into account the correct fluctuation matrix structure, but not the magnitude of fluctuations, with the previous method were only a diagonal D was used.

**Fig. 2:**
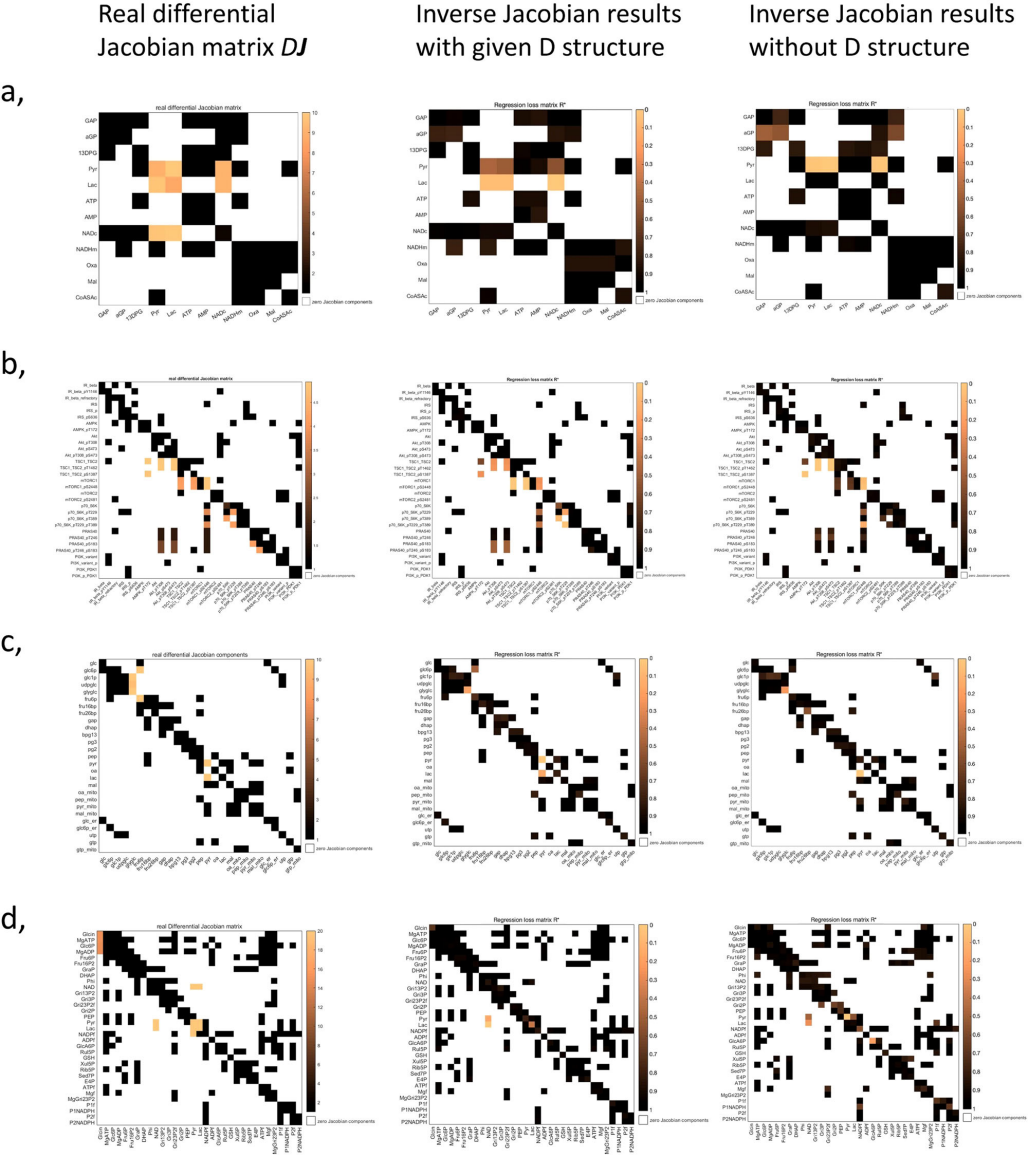
The regression loss Jacobian algorithm evaluation with or without D structure information. The evaluation models are: a, carbohydrate energy metabolism model^65^; b, AMPK-mTOR pathway model^66^; c, hepatic glucose metabolism model^67^ and d, blood cell metabolism model^68^. For each model, we generate Jacobian matrices for two conditions by changing reaction parameters, as describe in Supplementary Note 1. We apply random fluctuations directly to metabolites (diagonal fluctuation components) and to ¼ of the reactions (off-diagonal components). Inference of differential Jacobians is done according to the evaluation workflow. For each subplot a-d, left is the real differential Jacobian matrix; middle is the inverse differential Jacobian results when using the structure of D in the inference; and right is the result without D structure information for the inference, assuming a diagonal D. In each matrix heatmap, large values represent large differential Jacobian values, indicating large changes in interaction kinetics between the two conditions.

For this evaluation, we make use of the four models described in the Methods section. For each model, we generate artificial covariance matrix data with fluctuations applied to metabolites directly (diagonal fluctuation components) and ¼ of the reactions (off-diagonal components), where these fluctuations had the same magnitude. The covariance matrices for the two conditions are computed through the Lyapunov equation with *ε*_*D*_ = 0.4. As shown in Fig. 2, the original inverse differential Jacobian algorithm is impaired by the off-diagonal fluctuations. Using the structure information regarding the off-diagonal fluctuations, we achieve better results in matching the real differential Jacobian matrix. A more comprehensive evaluation of the accuracy is described in the next sections.

### Accuracy of the inverse Jacobian algorithm for different fluctuation magnitudes

**Fig. 3:**
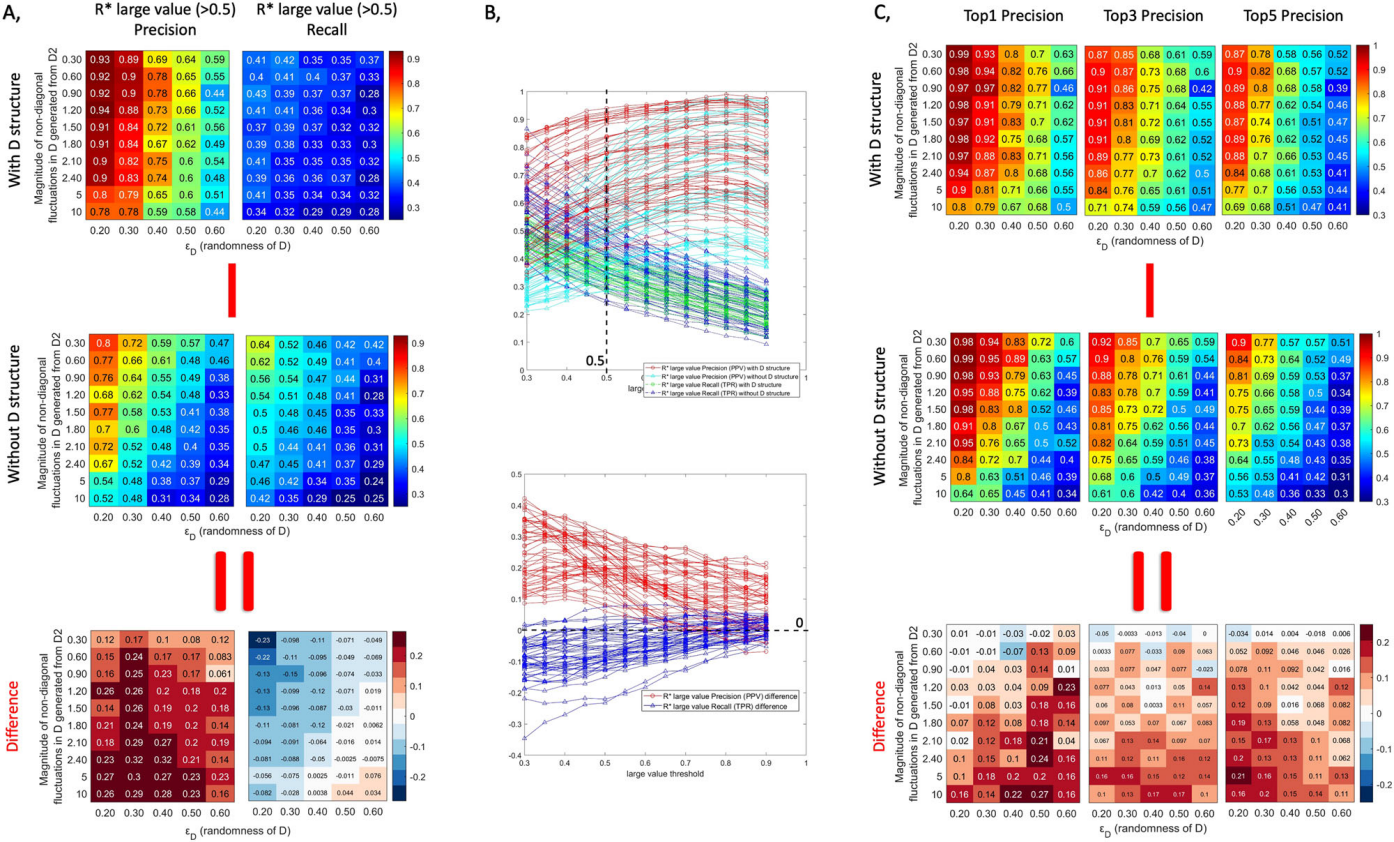
Inverse differential Jacobian algorithm with/without D structure over a range of fluctuation magnitudes (overall magnitude *ε*_*D*_ and magnitude ratio of off-diagonal fluctuations). The evaluation is conducted using the first model with 200 repeats and 3 random enzyme fluctuations applied. A, Precision and Recall of the large values (above 0.5) in ${{\boldsymbol{R}}}^{* }$ and *D****J*** over 200 repeats with/without D structure information; B, the line plots of Precision and Recall of the large values in ${{\boldsymbol{R}}}^{* }$ with/without D structure information based on different large value thresholds (0.3-0.9), where the snapshot of value 0.5 refers to A; C, the accuracy of the top 1, top 3 and top 5 large values in ${{\boldsymbol{R}}}^{* }$ over 200 repeats with/without D structure information. The bottom plots in A and C show the difference between top and middle plots.

To evaluate the effect of varying fluctuation magnitudes on the inverse Jacobian algorithm, we generated artificial covariance data for model 1 (carbohydrate energy metabolism) assuming a range of covariance values *ε*_*D*_ in the fluctuation matrix D, ranging from *ε*_*D*_ = 0.2 to *ε*_*D*_ = 0.6, and a relative magnitude of off-diagonal fluctuations Md ranging from 0.3 to 2.4. In this analysis, we apply non-diagonal fluctuations to three randomly chosen reactions, and we conduct a second test with six non-diagonal fluctuations applied, which yielded similar results as shown in Supplementary Fig. 2. For these data, the inverse Jacobian algorithm has been applied either without any structure information on D, or with assuming the correct structure as described in the methods section. Figure 3 presents the evaluation results, with the first two rows in Fig. 3A, C present the inverse Jacobian results without and with D structure respectively, and the third row displaying the difference between the second and the first rows. For each test with specific *ε*_*D*_ and off-diagonal fluctuation magnitude Md, we repeat the workflow 200 times. We evaluate the accuracy of finding correctly the large entries of the differential Jacobian by considering the resulting regression loss matrix R* over the 200 repeats. In Fig. 3A, B, and Supplementary Fig. 1, we compare the precision and recall of correctly identifying differential Jacobian values above a certain threshold, with or without D structure. As an alternative accuracy measure, in Fig. 3C, we compare the precision of correctly identifying the top 1, 3 and 5 values of the differential Jacobian through large entries in the regression loss matrix R*.

From the results, we can conclude that using the structure of the fluctuation matrix D will significantly improve the precision of identifying large values in the differential Jacobian. However, a surprising result is that using the correct D structure will decrease the recall of the large differential Jacobian values compared to just using a diagonal D. This conclusion remains similar when using different thresholds to classify large values as shown in Fig. 3A (threshold 0.5), Supplementary Fig. 1 (threshold 0.3 & 0.7) and 3B (line plots across thresholds 0.3–0.9). This tells us that using the correct fluctuation matrix structure will lower the hit rate, but enable to find the large values in the differential Jacobian with a much higher accuracy. Moreover, the precision of the resulting top 1, 3, and 5 interactions in the inverse Jacobian approach is always improved when using the correct structure of the fluctuation matrix D. Overall, this indicates that using the correct D structure may have more false negatives than the diagonal approach, but its hits will more reliably be true positives. This is consistent to the results in the breast cancer case study, where compared to assuming a diagonal D, the inverse Jacobian results using the D structure inferred from the metabolic reaction network will highlight fewer interactions with large values, while attributing a lower effect to the other interactions.

Moreover, the results shown in Fig. 3A, C suggest that a larger magnitude of covariance values in D has a detrimental effect on the inverse differential algorithm in both cases, with the correct structure and with only assuming a diagonal structure. The precision of the results decreases with an increase in the magnitude of non-diagonal fluctuations. However, the precision decrease is much smaller when using the correct structure of D. Subsequently, the results in the difference plot demonstrate that ignoring the off-diagonal elements in the structure of the fluctuation matrix D generally reduces the reliability of the inverse Jacobian algorithm compared to using the correct structure. However, if the off-diagonal values are smaller than the diagonal values, the results between using the correct structure of D and a diagonal D are comparable.

Finally, we perform the evaluation for all four literature models, applying only a small magnitude of Md = 0.3 as off-diagonal fluctuations to all reactions, with an overall fluctuation magnitude *ε*_*D*_ = 0.4, and do the inverse Jacobian analysis assuming a diagonal D.

### Accuracy of the inverse Jacobian algorithm with varying number and magnitude of off-diagonal fluctuations

In this section, we analyze the effect of additional assumptions of the fluctuation properties on the accuracy of the Jacobian reconstruction: the magnitude of off-diagonal fluctuations ranging from 0.3 to 10, and the number of off-diagonal fluctuations ranging from 6 to 42. This analysis is applied to the first evaluation model. Instead of perturbing reaction parameters, randomly chosen off-diagonal fluctuations are directly added to the fluctuation matrix D. This approach covers all possible non-diagonal fluctuations, not only those from enzyme activities, and can give us a better general understanding of the effect of non-diagonal fluctuations.

**Fig. 4:**
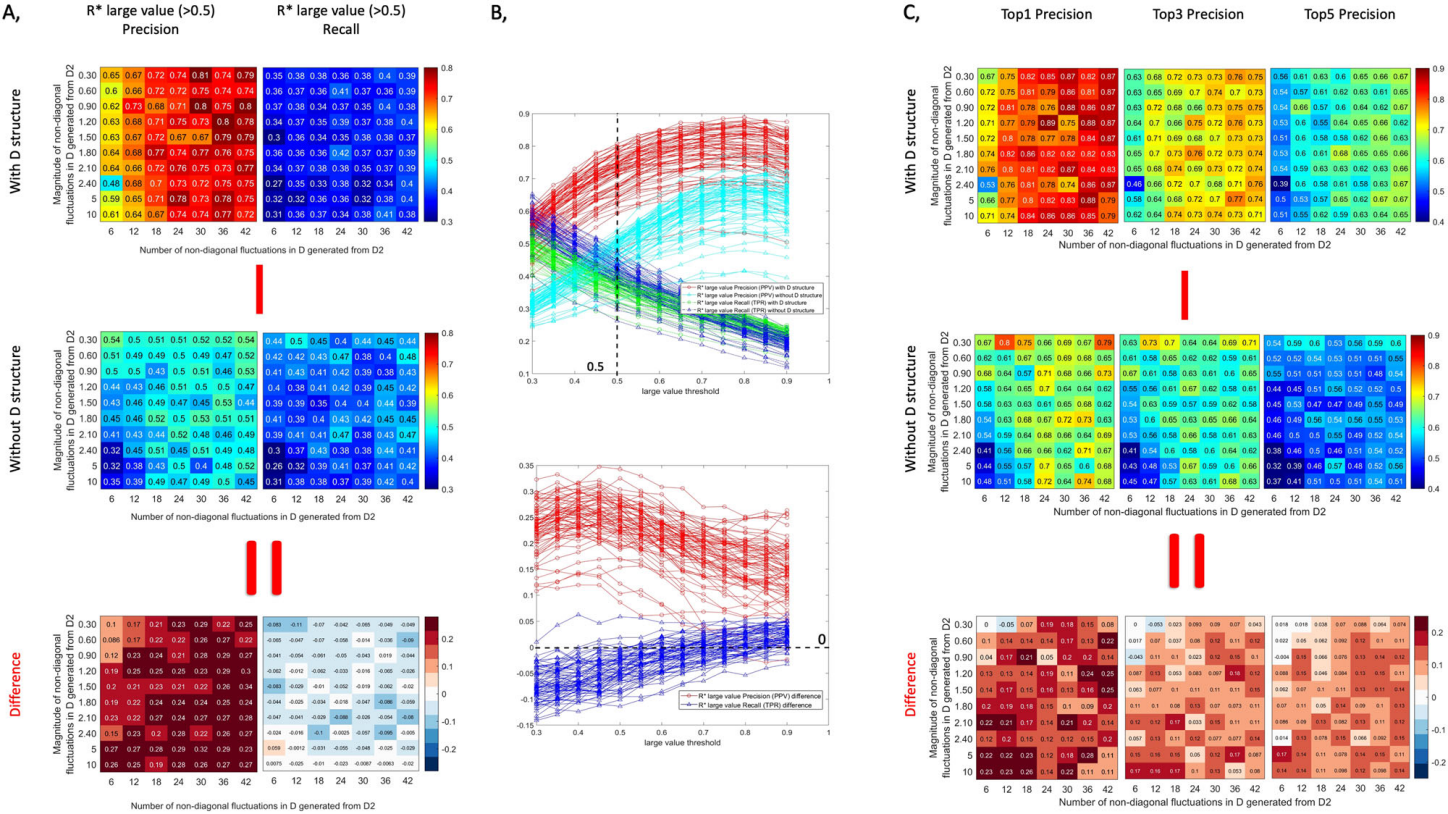
Inverse differential Jacobian algorithm results with/without D structure using a varying number and magnitude of off-diagonal fluctuations. The evaluation is conducted using model 1 form the methods section with 200 repeats and *ε*_*D*_ = 0.4. A, Precision and recall of the large values (above 0.5) in ${{\boldsymbol{R}}}^{* }$ and *D****J*** over 200 repeats with/without D structure information; B, the line plots of precision and recall of the large values in ${{\boldsymbol{R}}}^{* }$ with/without D structure information based on different large value thresholds (0.3-0.9), where the snapshot of value 0.5 refers to A; C, the accuracy of the top 1, top 3 and top 5 large values in ${{\boldsymbol{R}}}^{* }$ over 200 repeats with/without D structure information. The bottom plots in A and C are the difference between top and middle plots.

Using a similar evaluation as in the previous section, we compare the precision and recall of the large values in the inverse Jacobian over 200 repeats. The results are shown in Fig. 4 and Supplementary Fig. 4. As observed before, using structure information during the inference for the fluctuation matrix increases precision and decreases recall across various magnitudes and numbers of off-diagonal fluctuations compared to inverse Jacobian results assuming only a diagonal structure. When doing the inference with a diagonal fluctuation matrix, the inverse Jacobian algorithm accuracy remains relatively consistent across different numbers of small non-diagonal fluctuation, yet, increases with the introduction of larger non-diagonal fluctuations as seen in Fig. 4A, C and Supplementary Fig. 4. This is because the non-diagonal components in D originate from the same fluctuations added to several metabolites; consequently, these same fluctuations will also contribute to the related diagonal part of D. Thus, when the number of these fluctuations is large enough, the diagonal part becomes much larger due to the summation of several off-diagonal fluctuations. This further supports our conclusion that assuming a diagonal D remains feasible with only small off-diagonal fluctuations. For an additional validation, we conduct a similar evaluation with *ε*_*D*_ = 0.2 (compared to *ε*_*D*_ = 0.4 in Fig. 4), yielding consistent results as presented in Supplementary Fig. 5.

### Integrating fluctuation data further enhances the inverse Jacobian algorithm

**Fig. 5:**
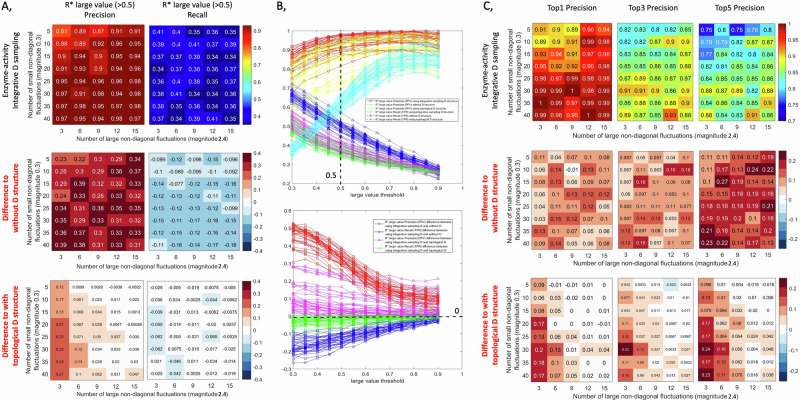
Inverse differential Jacobian results using various number of large (magnitude: 2.4) and small (magnitude: 0.3) non-diagonal fluctuations from three fluctuation matrix assumptions: 1, enzyme-activity integrative D sampling, 2, diagonal D and 3, topological D, middle/bottom plot in A and C is the difference In results between assumption 1 and assumption 2/3, respectively. The evaluation is conducted using model 1 from the methods section with 200 repeats and *ε*_*D*_ = 0.2. A, Precision and recall of the large values (above 0.5) in ${{\boldsymbol{R}}}^{* }$ and *D****J*** over 200 repeats with/without D structure information; B, the line plots of Precision and Recall of the large values in ${{\boldsymbol{R}}}^{* }$ with/without D structure information based on different large value thresholds (0.3-0.9), where the snapshot of value 0.5 refers to A; C, the accuracy of the top 1, top 3 and top 5 large values in ${{\boldsymbol{R}}}^{* }$ over 200 repeats with/without D structure information.

As described in the methods section, enzyme activity data can be set as upper constraints to conduct a more precise sampling of fluctuation values than the purely topology-based sampling used for the previous evaluations. To test the effect of taking this information into account, we vary the number of non-diagonal fluctuations, mixing among fluctuations of small (0.3) and large (2.4) magnitude. Using an approach as in Fig. 3, we evaluate the inverse Jacobian algorithm using three sampling strategies: a diagonal D, the topological D structure derived from considering only elements for large fluctuations, and integrative D sampling from enzyme expression or activity data, setting constraints on all fluctuations. Figures 5A, C and Supplementary Fig. 6 illustrate that the integrative D sampling strategy significantly improves the algorithm’s precision compared to using only the topological D structure, when the number of large fluctuations is comparatively small. For a comprehensive evaluation, we also conducted this analysis for a wider spread of fluctuation magnitudes with 10 vs. 0.3, and *ε*_*D*_ values of 0.2 and 0.4, respectively. Supplementary Figs. 7 & 8 present the results for these scenarios, which are in line with the results shown in Fig. 4.

### Application to a breast cancer dataset

Finally, we apply the inverse Jacobian approach to a breast cancer dataset from two cell lines^54^: a non-tumorigenic breast epithelial cell line (MCF102A) and a pleural effusion metastasis of a breast adenocarcinoma (MCF7). The objective is to find large values in the differential Jacobian between these cell lines from metabolomics covariance data. In addition to metabolites, the authors also measured transcriptomics data, and provided a genome-scale metabolic network model in which the reactions are annotated with gene IDs corresponding to the identifiers used in the transcriptomics data. Using the Cobra toolbox^55^, we are able to generate from the transcriptomics data a value for each reaction to represent the enzyme activity for that reaction. Collecting the enzyme activity profiles for the entire genome-scale model, we can then compute the variance of each enzyme activity separately for the two different cell lines. The histograms illustrating the variance in enzyme activity values for the two cell lines can be found in Supplementary Fig. 9. Notably, there are a significant portion of enzymes with small fluctuations, while only a very limited number of enzymes exhibit activity variances exceeding 200. Even though transcript levels are not fully representative of enzyme activity, we propose that it is a good proxy in our application, as we only use this value as an upper bound on relevant fluctuations instead of a definite value during the integrative sampling.

**Fig. 6:**
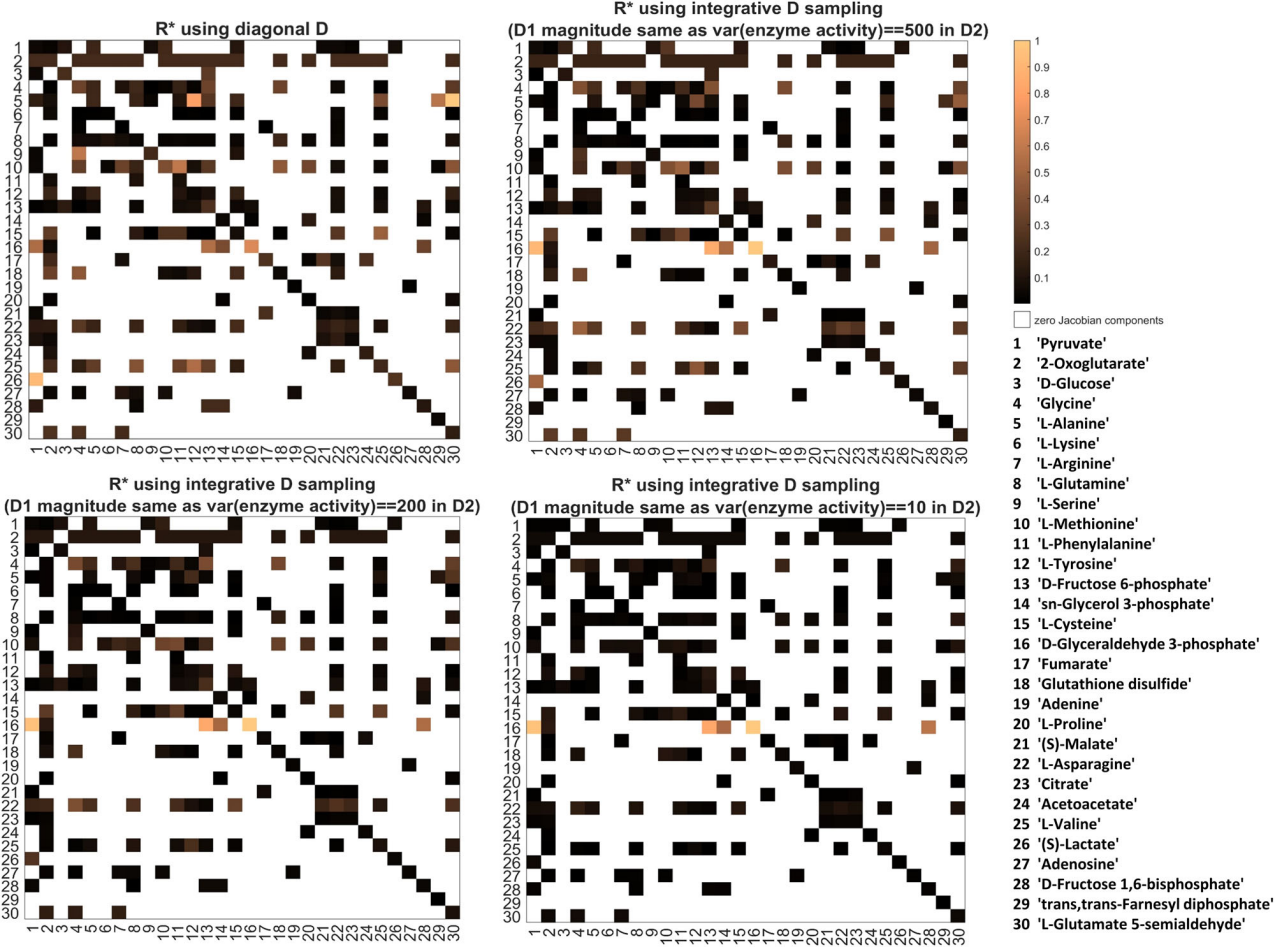
Inverse differential Jacobian analysis on the breast cancer dataset. The four panels show inference results using a diagonal D, and three sets of enzyme-activity based D_h_ and D_d_ sampling for inference with a non-diagonal D, where the magnitude of diagonal fluctuations defining D1 is scaled equal to fluctuations from enzyme activities with variances of 500, 200, and 10, respectively, defining D2.

Using the default setting in COVRECON toolbox, we first construct the metabolic interaction network for the metabolomics dataset. The resulting interaction structure is reported in Supplementary data 1. Consequently, we map the calculated variances of the enzyme activities to the determined metabolic interactions and use them to construct the upper bounds on the fluctuation matrix D as shown in Supplementary Fig. 10. We perform the inverse differential Jacobian algorithm with the integrative fluctuation sampling strategy. We compare different scaling of the fluctuations vs. each other by setting the magnitude of fluctuations affecting metabolites directly (*D*_1_) equal to fluctuations from enzyme activity (*D*_2_) with variance values equal to 500, 200, and 10, respectively. The results are then compared to the results obtained in our previous study using a diagonal D^30^, as depicted in Fig. 6.

It is apparent that when scaling the magnitude of diagonal fluctuations to be the same as a large non-diagonal enzyme activity fluctuation in D, with a variance of 500, the inverse Jacobian algorithm result with our new approach is close to the original inverse Jacobian algorithm result using a diagonal D sampling, as shown in right top subplot in Fig. 6. This result assumes that fluctuations acting directly on metabolites instead of on reactions are most important. Conversely, when scaling the magnitude of diagonal fluctuations equal to a small non-diagonal enzyme activity fluctuation with variance of 10, the results from the two approaches differ significantly. In this scenario, enzymes fluctuations play the dominant role. In line with the reduced recall seen in the previous evaluations, fewer, but more pronounced metabolite interactions are inferred to take a large value in the differential Jacobian. Following the increased precision of the enzyme-activity constrained inference seen in the previous tests, we postulate that these elements more reliably correspond to an actual change in metabolic interactions between the considered cell lines, even if from the available data one can not determine the actual ratio between fluctuations acting directly on metabolites and those acting on enzyme activities. The inverse Jacobian results show that the main dynamic difference between the cell lines lies on the metabolite D-Glyceraldehyde 3-phosphate, which is also identified as a biomarker in their original t-test^54^. Several interactions are identified as differences between the two cell lines where relevant enzymes activity also show significant difference^30^. The circular plots where one can check the superpathway information interactively are shown in Supplementary Fig. 11 and available as Matlab figure format in Supplementary data 1.

Unlike the changes in single metabolites identified through conventional statistical analysis, our approach reveals additional insights into the changes occuring at dynamic metabolic interactions between the two cell lines. The large dynamic differences between the two cell lines are identified as the high-valued elements in the right-bottom result of Fig. 6. From that inference, the highest changed interactions are pyruvate→ D-Glyceraldehyde 3-phosphate and D-Fructose 6-phosphate→ D-Glyceraldehyde 3-phosphate (see Supplementary Data 1 for numerical values). Further investigation of these interactions reveals that the enzyme transketolase (EC 2.2.1.1) is involved in both interactions. In fact, this enzyme has been widely found to play a vital role for the metabolic network switch of breast cancer^56–59^, and from the results of our analysis is predicted to also underly the metabolic differences between the two cell lines in this dataset.

Some of the interactions which we have identified as being highly changed between the cell lines with the different inverse differential Jacobian approaches could have been suspected by just looking at the enzyme fluctuation data (Supplementary Fig. 10). Specifically, the interactions sn-Glycerol 3-phosphate->D-Glyceraldehyde 3-phosphate and Pyruvate->(S)-Lactate are already assigned high enzyme fluctuation values. However, the interaction Pyruvate->(S)-Lactate is not seen in the inference with the non-diagonal D, so it might be a false positive from the previous algorithm.

Also, the enzyme fluctuations in each of the cell lines individually need of course not actually be related to changes between the cell lines. For this, one might look at the difference in enzyme fluctuations which we considered in Supplementary Fig. 12 (based on transcriptomics data). Even though the largest differences are related to Glutathione disulfide, this metabolite does not seem to be involved in highly changed dynamic interactions from the inverse Jacobian approaches. This suggests that the key changes in metabolic dynamics observed between the cell lines do rather stem from the effect of enzyme fluctuations on the reaction network, than absolute enzyme abundance inferred from transcriptomics, which supports taking the metabolic network structure into account as we are proposing in the integrative approach.

## Discussion

Jacobian analysis is an important approach for metabolic networks. Previous studies inferred kinetics, and the associated Jacobian matrix, with fluxomics data^33,34^ or time-series metabolomics measurements^35,36^. Using high-throughput metabolomics data samples from steady state, coupled with metabolic reaction databases, several studies have developed inverse Jacobian approaches^6,25,30,31,42,45^. In this article, we have enhanced this approach by leveraging more information about the structure of the fluctuation matrix, including reaction network structure and enzyme activity data. A novel fluctuation sampling strategy is applied by setting fluctuation constraints using variance in enzyme activity estimated from suitable data. We assessed the influence of these non-diagonal fluctuations on the inverse Jacobian algorithm based on three key factors: (1) the magnitude of covariance values, (2) the number of non-zero off-diagonal components in the fluctuation matrix D, and (3) the magnitude of off-diagonal components compared to that of diagonal components. The main findings are:

1. While sacrificing the positive hit rate, incorporating the non-diagonal D structure into the analysis significantly enhances the precision of the inverse Jacobian algorithm. Using an enzyme-activity based sampling strategy further improves the algorithm’s accuracy compared to using only a topological structure of the fluctuation matrix.
2. There are two cases in which assuming a diagonal fluctuation structure remains feasible for the inverse differential Jacobian analysis: (1) When non-diagonal fluctuations are relatively small in comparison to direct metabolite fluctuations; (2) when there are numerous non-diagonal fluctuations with similar magnitudes.

The inverse Jacobian approach aims to find changes in the causal dynamic regulations between two different metabolic conditions from measured covariance matrices, using extra information about the underlying network structure obtained from metabolic network databases. This new approach integrates fluctuation analysis and different OMICS datasets into the inverse Jacobian analysis, contributes to our deeper understanding of metabolic network interaction and dynamics, and enhances the robustness of the inverse Jacobian inference. Notably, without enzyme fluctuation data, this approach still improves the original algorithm by using only the topological structure of the fluctuation matrix. Meanwhile, several limitations still exist. Firstly, by trading off fluxomics and time-series measurements, this approach will need a large sample number (tens to hundreds) to achieve a relatively accurate covariance calculation. In addition, any allosteric interactions are not included in the considered network structure, due to lack of comprehensive knowledge available in databases.

The proposed algorithm is validated first by artificial data, and also by being able to show that a metabolic switch in breast cancer which was already known from other studies also underlies metabolic differences between the cell lines in the considered experimental dataset.

## Methods

### The differential Jacobian

Consider a biological system that consists of n metabolites denoted by ${\left\{{{\rm{X}}}_{{\rm{i}}}\right\}}_{{\rm{i}}=1\ldots {\rm{n}}}$. The vector M = {M_i_} = {|X_i_|} represents the concentrations of the n metabolites. The system dynamics can be modeled with the set of ordinary differential equations (ODEs):

1 $$\frac{d{\boldsymbol{M}}}{{dt}}={\boldsymbol{F}}\left({\boldsymbol{M}},{\boldsymbol{p}}\right)={\boldsymbol{S}}\,\cdot\, {\boldsymbol{v}}\left({\boldsymbol{M}},{\boldsymbol{p}}\right)$$

where ***S*** is the stoichiometry matrix formed by the stoichiometric coefficients of all the m reactions ${\boldsymbol{v}}\left({\boldsymbol{M}},{\boldsymbol{p}}\right)=\left[{v}_{1}\left({\boldsymbol{M}},{\boldsymbol{p}}\right),\cdots ,{v}_{m}({\boldsymbol{M}},{\boldsymbol{p}})\right]$ in the system. The reaction rates ${v}_{i}\left({\boldsymbol{M}},{\boldsymbol{p}}\right),i=1,...,m$ are usually modeled by Michaelis-Menten kinetics^60^ or mass action equations^61^ depending on metabolite concentrations ***M*** and parameters (e.g., enzyme activities) ***p***.

The steady-state Jacobian matrix ***J*** of Eq. (1) is defined as a |*R*^*n×n*^| matrix in which *J*_*ij*_ is the first-order derivative of the rate of change *f*_*i*_ of the metabolite concentration *M*_*j*_, evaluated at steady state, noted as ${J}_{{ij}}={\left.\frac{\partial {f}_{i}}{\partial {M}_{j}}\right|}_{{steady}}$:

2 $${\boldsymbol{J}}={\frac{\partial {\boldsymbol{F}}}{\partial {\boldsymbol{M}}}}_{{steady}}={\boldsymbol{S}}\,\cdot\, \left[\begin{array}{ccc}\begin{array}{cc}\frac{\partial {v}_{1}}{\partial {M}_{1}} & \frac{\partial {v}_{1}}{\partial {M}_{2}}\\ \frac{\partial {v}_{2}}{\partial {M}_{1}} & \frac{\partial {v}_{2}}{\partial {M}_{2}}\end{array} & \cdots & \begin{array}{c}\frac{\partial {v}_{1}}{\partial {M}_{n}}\\ \frac{\partial {v}_{2}}{\partial {M}_{n}}\end{array}\\ \vdots & \ddots & \vdots \\ \begin{array}{cc}\frac{\partial {v}_{m}}{\partial {M}_{1}} & \frac{\partial {v}_{m}}{\partial {M}_{2}}\end{array} & \cdots & \frac{\partial {v}_{m}}{\partial {M}_{n}}\end{array}\right]{steady}$$

In a previous study, Steuer et al. ^25^ used a model perturbed by stochastic fluctuations to establish a relation between the covariance matrix *C* of the metabolite concentrations and the steady-state Jacobian matrix of the system ***J*** given by the so-called Lyapunov equation

3 $${\boldsymbol{J}}\,\cdot\,C+C\,\cdot\,{{\boldsymbol{J}}}^{T}=-2D$$

Thereby, $C\in {R}^{n\times n}$ is the covariance matrix of the metabolite concentrations *M*_*j*_ around their steady-state values ${M}_{j}^{{steady}},{C}_{{ij}}=E\left[{M}_{i}{M}_{j}\right]-E[{M}_{i}]\,\cdot\, E[{M}_{j}]$, where *E* denotes the expected value. The fluctuation matrix *D* is the covariance of fluctuation sources acting on the system dynamics.

A typical inverse task in this setup is to infer the Jacobian matrix J, representing the interactions in the network, from estimates for C and potentially D from steady-state metabolomics data. Furthermore, for the inverse differential Jacobian, we more specifically infer the ratio between the Jacobian matrices for two biological conditions, for example a healthy and disease condition, abbreviated as ‘h’ and ‘d’, thereby identifying regulations in biochemical interactions that act differently between these two conditions. The differential Jacobian *D****J*** is defined as^30^:

4 $${D{\boldsymbol{J}}}_{{ij}}=\left\{\begin{array}{c}\max \left(\left|\frac{{({{\boldsymbol{J}}}_{{\boldsymbol{d}}})}_{{\boldsymbol{ij}}}}{{{({\boldsymbol{J}}}_{{\boldsymbol{h}}})}_{{\boldsymbol{ij}}}}\right|,\left|\frac{{({{\boldsymbol{J}}}_{{\boldsymbol{h}}})}_{{\boldsymbol{ij}}}}{{{({\boldsymbol{J}}}_{{\boldsymbol{d}}})}_{{\boldsymbol{ij}}}}\right|\right)\\ 1,{{{\boldsymbol{if}}({\boldsymbol{J}}}_{{\boldsymbol{h}}})}_{{\boldsymbol{ij}}}={\bf{0}}.\end{array}\right.$$

with the two conditional Jacobian matrix at steady-state denoted as ***J***_***h***_ and ***J***_***d***_.

The initial problem in the inverse task is that the system is under-determined, since the covariance matrix C and the fluctuation matrix D are symmetric matrices, but the Jacobian matrix ***J*** is in general not symmetric. Sun and Weckwerth addressed this problem by constraining the structure of the inferred Jacobian matrix from a topological metabolic interaction network, yielding entries which are constrained to zero in the Jacobian matrix ***J***^31^. They argued that the topological network can be built from a genome-scale network reconstruction, available from publicly accessible databases, such as KEGG^28^ and BioCyc^62^. At that time, the proposed algorithm was limited by two issues: they relied on structural network information that needs to be assembled manually, and they encountered a numerical instability problem due to ill-conditioned regression problems for large-scale metabolic networks^30,45^. In a more recent study, we developed a fully automated COVRECON workflow and related Matlab toolbox^30^ that can automatically construct the topology of the metabolic interaction network from KEGG^28^, BiGG^27^ and ModelSeed^29^ databases, and replaces the ill-conditioned regression problem with a regression loss-based inverse Jacobian algorithm.

### Inferring the fluctuation matrix structure and adding constraints based on enzyme activity data

Previous inverse Jacobian algorithms^25,30,31,44,45^ have assumed that independent stochastic noise affects each metabolite individually, giving rise to a diagonal fluctuation matrix D in the Lyapunov Eq. (3). In this article, we introduce additional stochastic fluctuations in enzyme activity^47,48,50,51^. Through the coupling of metabolites by reactions, these result in correlated fluctuations of metabolite concentrations. Importantly, we assume that noise acting through the enzyme activity and noise acting directly on metabolites are statistically independent noise sources.

Suppose in the same system with dynamics Eq. (1), the steady-state concentration of *X*_*i*_ ${\rm{is}}$ ${\boldsymbol{M}}=\left[{M}_{{\boldsymbol{i}}}^{{\boldsymbol{0}}}\right]$, when the parameters ***p*** take the nominal values ***p***_**0**_. We consider stochastic fluctuations on the metabolites as Gaussian noise vector ***ω***, and stochastic fluctuations of parameters ***p*** with a Gaussian noise vector ***τ***. According to the above assumption, ***ω*** and ***τ*** are statistically independent. Thus, the reaction parameter vector becomes ${\boldsymbol{p}}\left(t\right)={{\boldsymbol{p}}}_{{\boldsymbol{0}}}+{\boldsymbol{\tau }}$. Small fluctuations of metabolite concentrations around steady state are described by

5 $${M}_{i}={M}_{i}^{0}+{m}_{i}$$

The vector ${\boldsymbol{m}}={\boldsymbol{[}}{m}_{i}]$ is the variation of metabolite concentrations around steady-state. Overall, the dynamics with stochastic fluctuations thus become

6 $$\frac{d{\boldsymbol{M}}}{{dt}}={\boldsymbol{S}}\,\cdot\, {\boldsymbol{v}}\left({\boldsymbol{M}},{\boldsymbol{p}}\right)+{\boldsymbol{\omega }}={\boldsymbol{S}}\,\cdot\, {\boldsymbol{\nu }}\left(\left({{\bf{M}}}^{{\bf{0}}}+{\boldsymbol{m}}\right),\left({{\boldsymbol{p}}}_{{\bf{0}}}+{\boldsymbol{\tau }}\right)\right)+{\boldsymbol{\omega }}$$

A Taylor expansion near the system’s nominal steady state $({{\bf{M}}}^{{\boldsymbol{0}}},{{\boldsymbol{p}}}_{{\boldsymbol{0}}})$ then yields

$$\begin{array}{lll}\frac{d{\boldsymbol{m}}}{{dt}}&=& {\boldsymbol{S}}\,\cdot\, {\boldsymbol{\nu }}\left({{\bf{M}}}^{{\bf{0}}},{{\boldsymbol{p}}}_{{\bf{0}}}\right)+{\boldsymbol{S}}\,\cdot\, \left({\left.\frac{\partial {\boldsymbol{\nu }}}{\partial {\boldsymbol{M}}}\right|}_{{{\bf{M}}}^{{\bf{0}}},{{\boldsymbol{p}}}_{{\bf{0}}}}\,\cdot\, {\boldsymbol{m}}+{\left.\frac{\partial {\boldsymbol{\nu }}}{\partial {\boldsymbol{p}}}\right|}_{{{\bf{M}}}^{{\bf{0}}},{{\boldsymbol{p}}}_{{\bf{0}}}}\,\cdot\, {\boldsymbol{\tau }}\right)+{\boldsymbol{\omega }}\\ &=& {\boldsymbol{S}}\,\cdot\, {\left.\frac{\partial {\boldsymbol{\nu }}}{\partial {\boldsymbol{M}}}\right|}_{{{\bf{M}}}^{{\bf{0}}},{{\boldsymbol{p}}}_{{\bf{0}}}}\,\cdot\, {\boldsymbol{m}}+{\boldsymbol{S}}\,\cdot\, {\left.\frac{\partial {\boldsymbol{\nu }}}{\partial {\boldsymbol{p}}}\right|}_{{{\bf{M}}}^{{\bf{0}}},{{\boldsymbol{p}}}_{{\bf{0}}}}\,\cdot\, {\boldsymbol{\tau }}+{\boldsymbol{\omega }}\end{array}$$

Observing that ${\boldsymbol{K}}={\left.\frac{\partial {\boldsymbol{\nu }}}{\partial {\boldsymbol{p}}}\right|}_{{{\bf{M}}}^{{\boldsymbol{0}}},{{\boldsymbol{p}}}_{{\boldsymbol{0}}}}$ is a constant matrix, we get

$$\frac{d{\boldsymbol{m}}}{{dt}}={\boldsymbol{J}}\,\cdot\, {\boldsymbol{m}}+{\boldsymbol{S}}\,\cdot\, \left({\boldsymbol{K}}\,\cdot\, {\boldsymbol{\tau }}\right)+{\boldsymbol{\omega }}\triangleq {\boldsymbol{J}}\,\cdot\, {\boldsymbol{m}}+\Delta$$

Here ${\boldsymbol{\triangle }}={\boldsymbol{S}}\,\cdot\, \left({\boldsymbol{K}}\,\cdot\, {\boldsymbol{\tau }}\right)+{\boldsymbol{\omega }}$ is the total stochastic fluctuation acting on the metabolites. The stationary solution of *P* (***m***) of the previous equation is known to be a multivariate Gaussian distribution, and the corresponding derivation of Eq. (3) has been presented previously^25,63^. In our case, the fluctuation matrix D is determined as

7 $$\begin{array}{lll}{\rm{D}}={\boldsymbol{E}}[\Delta \,\cdot\, {\Delta }^{{\boldsymbol{T}}}]&=& {\boldsymbol{E}}[\left({\boldsymbol{S}}\,\cdot\, \left({\boldsymbol{K}}\,\cdot\, {\boldsymbol{\tau }}\right)+{\boldsymbol{\omega }}\right)\,\cdot\, {\left({\boldsymbol{S}}\,\cdot\, \left({\boldsymbol{K}}\,\cdot\, {\boldsymbol{\tau }}\right)+{\boldsymbol{\omega }}\right)}^{T}]\\ &=& {\boldsymbol{S}}\,\cdot\, {\boldsymbol{K}}\,\cdot\, {\boldsymbol{E}}[{\boldsymbol{\tau }}\,\cdot\, {{\boldsymbol{\tau }}}^{{\boldsymbol{T}}}]\,\cdot\, {{\boldsymbol{K}}}^{T}\,\cdot\, {{\boldsymbol{S}}}^{T}+E\left[{\boldsymbol{\omega }}\,\cdot\, {{\boldsymbol{\omega }}}^{{\boldsymbol{T}}}\right]\\ &=& {\boldsymbol{S}}\,\cdot\, \left({\boldsymbol{K}}\,\cdot\, {D}_{2}\,\cdot\,{{\boldsymbol{K}}}^{{\boldsymbol{T}}}\right)\,\cdot\, {{\boldsymbol{S}}}^{T}+{D}_{1}\end{array}$$

where ${D}_{1}={\boldsymbol{E}}{\boldsymbol{[}}{\boldsymbol{\omega }}{\boldsymbol{\bullet }}{{\boldsymbol{\omega }}}^{{\boldsymbol{T}}}]$ and ${D}_{2}={\bf{E}}{\boldsymbol{[}}{\boldsymbol{\tau }}{\boldsymbol{\bullet }}{{\boldsymbol{\tau }}}^{{\boldsymbol{T}}}{\boldsymbol{]}}$ are diagonal matrices describing the covariance of fluctuations acting on metabolite concentrations and reaction parameters, respectively.

Equation (7) gives the structure of the fluctuation matrix D in the Lyapunov Eq. (3), where the matrix *D*_1_ represents the covariance of stochastic fluctuations acting directly on metabolites, and *D*_2_ represents the covariance of stochastic fluctuations in the reaction rate parameters ***p***. According to our assumption that noise acting on the individual components is statistically independent, we can model *D*_1_ and *D*_2_ as diagonal matrices. In specific cases, coordinated gene regulation may also lead to correlated fluctuations in reaction rate parameters, which could be reflected by off-diagonal entries in *D*_2_. Furthermore, enzyme activity or expression data could be used to further constrain the entries of *D*_2_. As shown in Fig. 1, we claim that enzymes with large activity variances indicate large fluctuations in related reaction rate parameters ***p***.

### Superpathway based zero and sign structure of the fluctuation matrix

In the previous section, the structure of the fluctuation matrix D is derived for a complete metabolic network. However, in most experiments only a limited number of metabolites is measured which often not provides full coverage of the network. For that situation, we previously developed the COVRECON approach, where we construct a reduced metabolic interaction network for the measured metabolites based on superpathways. Each interaction is a superpathway that consists of several reaction steps taking all the possible interaction effects (reactant-product, reactant-reactant, product-product) into account^30,42^. Notably, each enzyme fluctuation within a superpathway will exert a correlated influence on the metabolites at both ends of the superpathway, introducing off-diagonal components into the structure of the fluctuation matrix D. Consequently, we can map the enzyme-reaction associations to the non-zero elements of D.

**Fig. 7:**
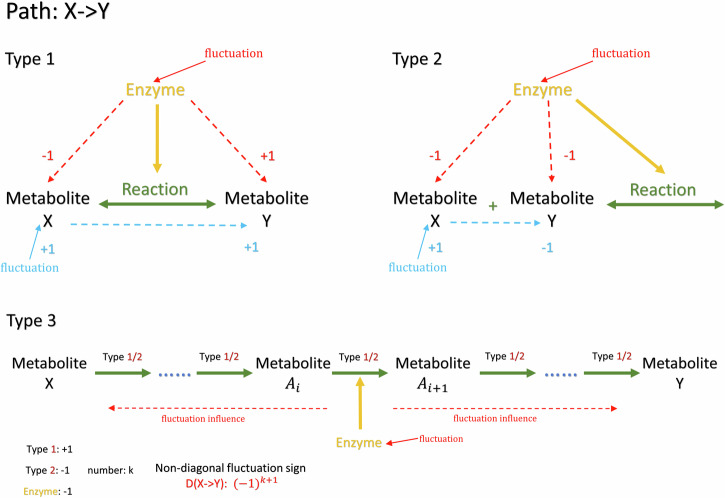
Determine the sign of the non-diagonal D structure in several cases. After generating the metabolic interaction network and the corresponding sparsity structure of matrices J and D, we are able to further determine the sign of the elements in the matrix D based on the related superpathway information according to three types. The non-diagonal element has a negative sign for type 1, and a positive sign for type 2. In the more complicated type 3, the non-diagonal D element has the sign ${-1}^{k+1}$, where k is the number of type 2 connections in the related superpathway.

The reaction structure also allows to distinguish positive and negative off-diagonal elements in D (due to covariance properties, the diagonal elements are all positive). Figure 7 illustrates the three types of superpathways that we need to consider. In the first two types, superpathway X- > Y is just a one-step pathway. Perturbations on this reaction rate will act positively on the products and negatively on the reactants. Thus, when X and Y are located on opposite sides of the reaction (type 1), the perturbation changes X and Y in different directions, leading to a negative sign in the corresponding element. Conversely, when X and Y are on the same side of the reaction (type 2), the perturbation affects both X and Y in the same direction, leading to a positive sign in the element of D corresponding to the interaction between X and Y. On the other hand, when introducing a perturbation directly to the metabolite instead of an enzyme, it will enhance the reaction rate and exert a decreasing effect on other metabolites on the same side. Moreover, the influence will pass through the other side of the reaction and influence the metabolites on the other side in the same way. In the more complicated type 3, the superpathway from X to Y consists of several steps of either of the first two types with a number of intermediate metabolites *A*_*i*_. Assuming the enzyme perturbation acts on intermediate step ${A}_{k}\to {A}_{k+1}$, it generates perturbations to both metabolites ${A}_{k}$ ${\rm{and}}$ ${A}_{k+1}$ following the scenarios in type 1 or 2. These perturbations propagate in both directions along the entire superpathway up to X and Y. As illustrated in Fig. 7, for the intermediate steps corresponding to type 1, that results in a negative sign; while for intermediate steps of type 2, it results in a positive sign. Consequently, if there are k steps of type 2 where *A*_*i*_ and *A*_*i*+1_ are on the same side of a reaction, the final sign of the D element corresponding to the interaction between X and Y will be ${-1}^{k+1}$.

The (sign) structure of the fluctuation matrix D is used in the COVRECON Jacobian algorithm to constrain the sampling range for the considered fluctuation values during the inference.

### COVRECON workflow and regression loss based differential Jacobian algorithm

Once we have the structure information of fluctuation matrix D, we follow the same workflow as outlined in COVRECON^30^. It consists of three sub-modules: (i) building of an organism-specific database, (ii) construction of a superpathway-based topological model for metabolite interactions using a pathway search based on the generated organism-specific database, (iii) the regression loss based inverse differential Jacobian computation. Thereby, in step (iii), we sample fluctuation matrices according to the structure of the fluctuation matrix as derived above.

In the inverse Jacobian approach, the Lyapunov Eq. (3) is solved using optimization with the data-based covariance matrix C and a sampled fluctuation matrix D. This linear equation can be rewritten in the form

8 $$\begin{array}{c}{{\rm{A}}}_{h}\,{{\rm{q}}}_{h}={{\rm{b}}}_{h}\\ {{\rm{A}}}_{d}\,{{\rm{q}}}_{d}={{\rm{b}}}_{d}\end{array}$$

where Eq. (3) can be transformed into the form Aq = b, A, q and b are computed from corresponding elements from C, J and D respectively^44,64^. Li, et al. ^30^ showed that under numerical variations in b, coming from sampled fluctuation values, the variation of the regression solution q is much larger compared to the variation in the regression loss *r*. Based on this property, we construct a “regression loss matrix” ${{\boldsymbol{R}}}^{* }$ that aims to capture the relative importance of individual elements in the differential Jacobian, rather than directly calculating the actual values in the differential Jacobian matrix. Each element of this regression loss matrix R^*^ is calculated by solving the linear Eq. (8) with an additional constraint, giving the solution

$${q}_{s}^{* }={{({A}_{c}}^{T}{A}_{c})}^{-1}{{A}_{c}}^{T}{b}_{c}$$

where *A*_*c*_ is constructed by combining *A*_*h*_ and *A*_*d*_ in Eq. (8) with the additional constraint that only element *J*_*ij*_ is the same between the Jacobians *J*_*h*_ and *J*_*d*_, and ${b}_{c}=[{b}_{h};{b}_{d}]$. If the assumption is incorrect, the element *J*_*ij*_ will be different for the two Jaocbian matrixes.

The regression loss with respect to that element *J*_*ij*_, corresponding to the element ${R}_{{ij}}^{* }$ of the regression loss matrix, is then defined as

9 $${R}_{{ij}}^{* }={\min }_{{b}_{c}}{||}{b}_{c}-{A}_{c}{q}_{s}^{* }{||}$$

Because we only have the structure information of the fluctuation matrixes *D*_*h*_ and *D*_*d*_, not the actual values, we iterate the regression loss over a number of samples for possible values of the fluctuation matrix that are generated according to its non-zero structure as derived above. The results reported in this paper are based on using 1000 samples. Then, the final ${R}_{{ij}}^{* }$ is taken as the minimum regression loss for different values of *b*_*c*_ in the different samples. If the above constraint on the Jacobian element *J*_*ij*_ is incorrect, the regression loss will increase, and thus a larger ${R}_{{ij}}^{* }$ reflects larger differences between *J*_*h*_ and *J*_*d*_. For simplicity, the elements in ${{\boldsymbol{R}}}^{* }$ are normalized to [0,1]. In this work, we use three different sampling methods of the fluctuation matrices *D*_*h*_, *D*_*d*_ and corresponding *b*_*c*_ for the inverse Jacobian approaches described in the following section.

In the resulting regression loss matrix ${{\boldsymbol{R}}}^{* }$, larger values indicate correspondingly larger values in the differential Jacobian matrix and large metabolic interaction changes between the two conditions. This relation has been shown in ref. ^30^, where also further details of our inverse differential Jacobian algorithm are described.

### Fluctuation sampling constraints based on enzyme activity data

In the first approach in this paper as well as in the original inverse Jacobian algorithms, the fluctuation matrix D is sampled according to the assumed structure (diagonal or with non-zero off-diagonal elements) with values from an arbitrary “normalized” range between 0 and 1. This assumes that no information on the magnitude of the fluctuations acting on the metabolic network is available.

Additionally, we propose a second integrative approach by applying constraints during sampling of the D matrix from variability in enzyme activity, where larger enzyme activity variance allows larger fluctuation in reaction rates. We integrate all the non-diagonal fluctuations inferred from the whole enzyme activity dataset, and sample each non-zero component in *b*_*c*_ with its absolute value between zero and the enzyme activity variance related to that component. Thereby, the sign of each *b*_*c*_ component is determined as in Fig. 7, or randomly given +1 or -1 if not clearly determined.

### Evaluation of differential Jacobian inference with literature models

To evaluate our algorithm and the potential effect of non-diagonal fluctuations on the differential Jacobian, we consider the four literature models that have also been used in our previous study^30^. Supplementary Note 1 gives the model references, and describes how we construct the two conditions required to define a differential Jacobian. The Jacobian matrices are available in Supplementary data 1. The resulting differential Jacobian matrices for these four cases are shown in Fig. 2, left column.

Similar as in previous studies^30,31,44,45^, we generate artificial perturbed covariance data for evaluation from the Lyapunov equation by giving random fluctuation matrixes *D*_*h*_ and *D*_*d*_ with different randomness *ε*_*D*_, see ref. ^30^. The structures of matrixes *D*_*h*_ and *D*_*h*_ are randomly generated as non-diagonal matrixes.

In the evaluation, we compare three inverse Jacobian algorithm:

1. The original COVRECON approach^30^, assuming a diagonal fluctuation matrix D.
2. With D structure: Assumes the structure information of D is known (given in evaluation models and constructed from COVRECON approach in general cases), where D elements have values of 0, +1 or −1.
3. Enzyme activity integrative D sampling: Assumes the structure information of D is known, with upper constraints on non-diagonal elements based on additional omics data (e.g., enzyme activity data).

### Workflow to evaluate the inference algorithm

**Fig. 8:**
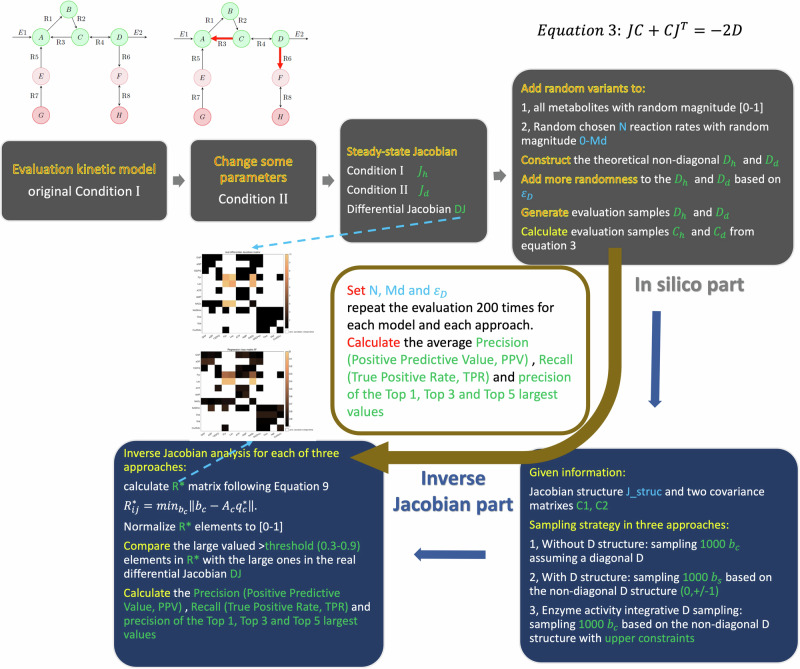
Evaluation workflow. For each evaluation model, we first generate two conditions as in Supplementary Note 1 and calculate the two Jacobian matrixes *J*_*h*_, *J*_*d*_ and the differential Jacobian matrix *D****J***. Then for each setting N, Md and *ε*_*D*_, we generate in silico *C*_*h*_ and *C*_*d*_ as described in the methods. In the inverse Jacobian part, we calculate R* from *C*_*h*_, *C*_*d*_, the Jacobian structure matrix, and different D structure constraints from the three approaches. Finally, we calculate several performance measures to represent the identification accuracy of the large elements in R* compared to the real *D****J***. For each setting N, Md and *ε*_*D*_, we repeat the random evaluation 200 times and calculate the average precision, recall of large elements, and accuracy of Top values in R* vs *D****J***.

The overall workflow for evaluation is shown in Fig. 8 and includes two parts: an in silico part to generate artificial data, and an inverse Jacobian part for the actual inference algorithm. First, for each evaluation model, we generate a second condition by changing some parameters (detailed in Supplementary Note 1). The Jacobian matrices of the two conditions (h and d) are determined from the given kinetic model, and the real differential Jacobian matrix *D****J*** is calculated as in Eq. 4. Subsequently, we generate in silico covariance matrixes *C*_*h*_ and *C*_*d*_. In previous studies we employed two methods for that: first, a numerical simulations of the underlying stochastic differential equation (SDE) to get the samples for two conditions, then calculate *C*_*h*_ and *C*_*d*_; second, to calculate *C*_*h*_ and *C*_*d*_ directly from solving Eq. 3 with sampled *D*_*h*_ and *D*_*d*_. We have shown previously that these two methods are essentially equivalent. However, the second method significantly saves on computation time^30,31,44,45^, which is why we generated artificial data with this method.

In the in silico part of this evaluation, we first sample fluctuation matrices *D*_*h*_ and *D*_*d*_ depending on three parameters: (1) the magnitude of the fluctuation covariance *ε*_*D*_, (2) the number of off-diagonal components in the fluctuation matrix D, denoted as N and (3) the fluctuation magnitude of off-diagonal components compared to the diagonal components in fluctuation matrix D, denoted as Md. First, N off-diagonal elements are randomly chosen to get non-zero values. Then, we assign a random magnitude in [0,1] to each (diagonal) element of D, and add a random magnitude in [0, Md] to each of the N non-zero off-diagonal entries and the corresponding diagonal element. To introduce variability that would stem from limited sampling size in real experiments, we finally add further random noise to all elements in *D*_*h*_ and *D*_*d*_, with standard deviation *ε*_*D*_, scaled to the magnitude of off-diagonal components Md, to Dh and Dd. Using the fluctuation matrices *D*_*h*_ and *D*_*d*_, we calculate in silico *C*_*h*_ and *C*_*d*_ by Eq. 3.

In the second part of the workflow, for the inference algorithm using the Lyapunov equation, we only make use of the structural information of the Jacobian matrix (elements valued 0 or 1), the considered constraints on the fluctuation matrix D, and the given data represented by the covariance matrices *C*_*h*_ and *C*_*d*_. We conduct the three inverse Jacobian approaches as described above, using 1000 samples for the fluctuation matrix, corresponding to *b*_*c*_ in (9). Finally, to evaluate the performance of the inference, we compare the large values in the regression loss matrix R* with the actual differential Jacobian *D****J*** known from the model.

The whole evaluation process is repeated 200 times for each choice of the parameters used in the generation of artificial data.

### Inverse differential Jacobian accuracy

To evaluate how accurately the large components in the real differential Jacobian matrix *D****J*** are identified, we assess the Precision (Positive Predictive Value, PPV) and Recall (True Positive Rate, TPR) of the matrix elements exceeding different threshold values (0.3–0.9) in the regression loss matrix ${{\boldsymbol{R}}}^{* }$. In addition, we assess the precision of the Top 1, Top 3 and Top 5 largest values by rank in ${{\boldsymbol{R}}}^{* }$.

### Enzyme activity upper constraints estimation using the Cobra toolbox

In the breast cancer dataset^54^, the transcriptomics data were measured, and a manually curated genome-scale model was provided with the manuscript (‘RECON3D_301_hgnc_id.xml’). We map the transcriptomics data to enzyme activities with the function ‘mapExpressionToReactions’ in the Cobra toolbox, using max for AND and sum for OR operators in the gene-protein-reaction-association.

## Supplementary information

Supplementary information

Supplementary data

## Data Availability

The data underlying this article are available in the online supplementary material. The original data of the breast cancer case study can be accessed in the Ref. ^54^

## Code availability

The Matlab code is available in https://bitbucket.org/mosys-univie/covrecon/
