# Supplementary material for: Network structure and fluctuation data improve inference of metabolic interaction strengths with the inverse Jacobian
Source: NPJ Syst Biol Appl. 2024 Nov 23;10:137. doi: 10.1038/s41540-024-00457-y (PMC11585569; doi:10.1038/s41540-024-00457-y)
Supplement: Supplementary file 1 — Supplementary information [file 41540_2024_457_MOESM1_ESM.pdf]

Supplementary Information:

Network structure and fluctuation data improve inference of metabolic interaction strengths with the inverse Jacobian

Contents

- Supplementary Note 1 2
- Supplementary Figure 1 3
- Supplementary Figure 2 4-5
- Supplementary Figure 3 6
- Supplementary Figure 4 7
- Supplementary Figure 5 8
- Supplementary Figure 6 9
- Supplementary Figure 7 10-11
- Supplementary Figure 8 12-13
- Supplementary Figure 9 14
- Supplementary Figure 10 15
- Supplementary Figure 11 16-17
- Supplementary Figure 12 18

Supplementary Note 1

Evaluation literature models

To evaluate our algorithm and the potential effect of non-diagonal fluctuations on the differential Jacobian, we consider the four literature models that have also been used in our previous study [1]. We describe how to generate the two conditional Jacobians as following:

1, A model of carbohydrate energy metabolism [2]: One condition is obtained from the nominal parameter values; for the other condition we increased the reaction rate parameter in R2, Pyrute+NADHc→Lactose+NADc, five-fold.

2, AMPK-mTOR pathway model [3]: The paper describes a wild type and an mTOR knockout model based on time-series experimental data; these two variants are used to define the differential Jacobian.

3, Hepatic glucose metabolism model [4]. The first condition is from nominal parameters. We applied a two-fold parameter change for the reaction rate of the second reaction R2 for the second condition.

4, Large-scale blood cell metabolism model [5]. We introduced a five-fold increase to several components of the Jacobian matrix directly.

These Jacobian matrixes are available in Supplementary data 1.

References

1. Li, J., S. Waldherr, and W. Weckwerth, *COVRECON: Automated Integration of Genome- and Metabolome-Scale Network Reconstruction and Data-driven Inverse Modeling of Metabolic Interaction Neworks.* Bioinformatics, 2023.
2. Nazaret, C. and J.-P. Mazat, *An old paper revisited:"A mathematical model of carbohydrate energy metabolism. Interaction between glycolysis, the Krebs cycle and the H-transporting shuttles at varying ATPases load" by VV Dynnik, R. Heinrich and EE Sel'kov.* Journal of theoretical biology, 2008. **252**(3): p. 520-529.
3. Dalle Pezze, P., et al., *A systems study reveals concurrent activation of AMPK and mTOR by amino acids.* Nature communications, 2016. **7**(1): p. 1-19.
4. Bulik, S., H.-G. Holzhütter, and N. Berndt, *The relative importance of kinetic mechanisms and variable enzyme abundances for the regulation of hepatic glucose metabolism–insights from mathematical modeling.* BMC biology, 2016. **14**(1): p. 1-22.
5. Holzhütter, H.G., *The principle of flux minimization and its application to estimate stationary fluxes in metabolic networks.* European journal of biochemistry, 2004. **271**(14): p. 2905-2922.

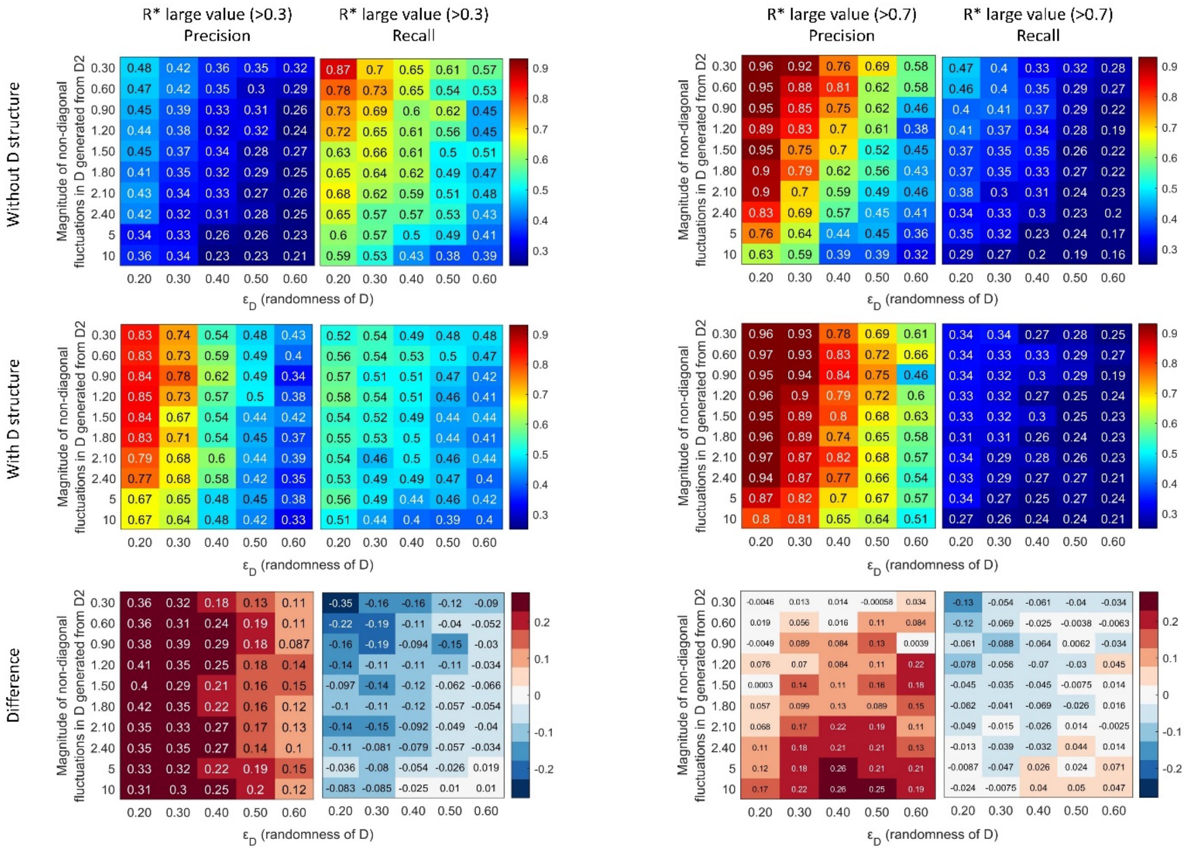

Supplementary Figure 1. Precision and Recall of the large values in R^* based on different thresholds (left: 0.3, right: 0.7). Color code refers to Figure 4A.

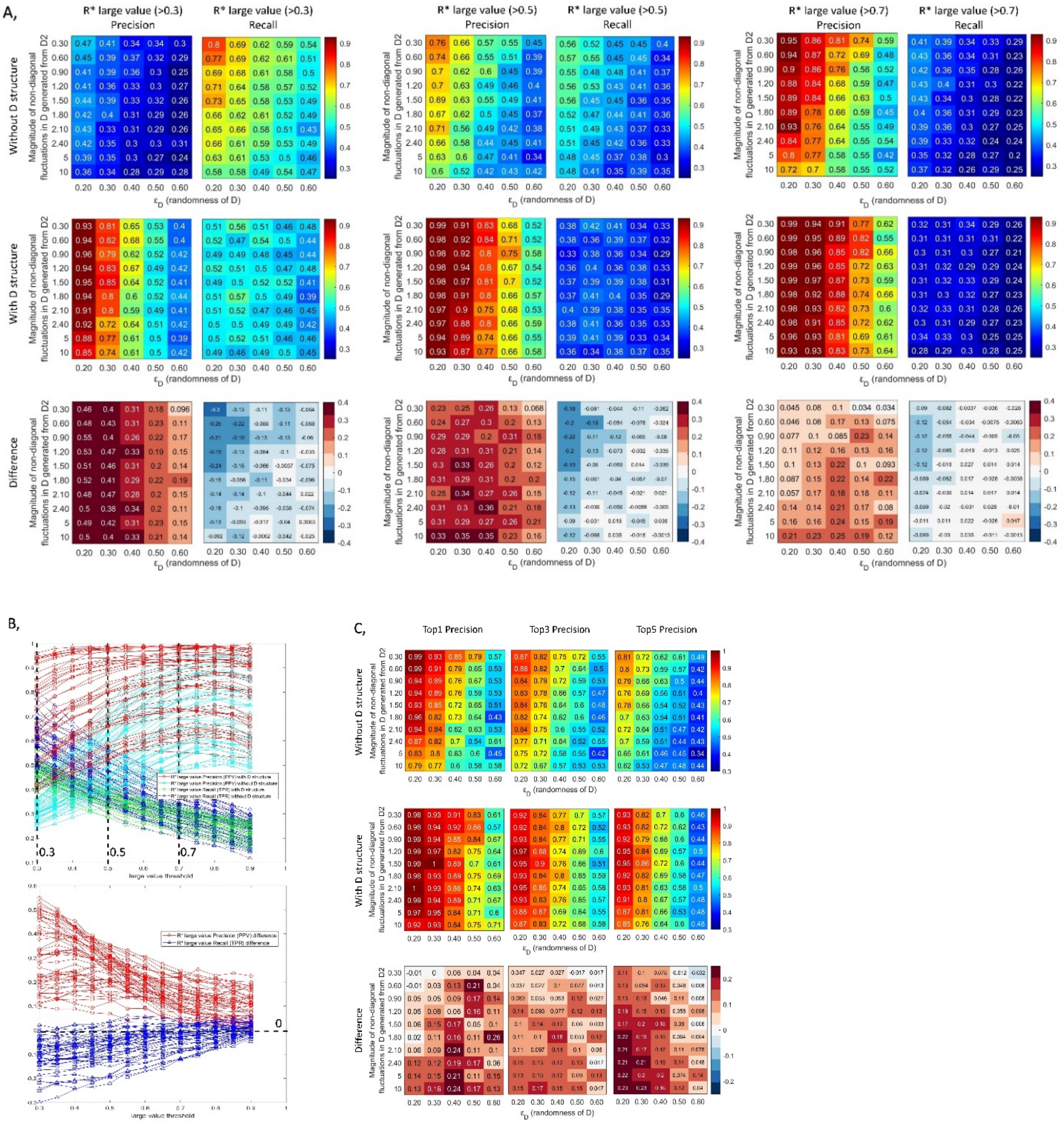

Supplementary Figure 2. Inverse differential Jacobian algorithm with/without D structure evaluation using various randomness of D ε_D and the magnitude of off-diagonal fluctuations. The evaluation is conducted using the first model in method section with 200 repeats and 6 random enzyme fluctuations applied. A, Precision and Recall of the large values (above 0.5) in $\boldsymbol{R}^*$ over 200 repeats with/without D structure information; B, the line plots of Precision and Recall of the large values in $\boldsymbol{R}^*$ with/without D structure information based on different large value thresholds (0.3-0.9), where the snapshots of value 0.3,

0.5 and 0.7 refer to A; C, the accuracy of the top 1, top 3 and top 5 large values in $\boldsymbol{R}^*$ over 200 repeats with/without D structure information.

Real differential Jacobian matrix *DJ*

All reactions perturbed with 0.3 times magnitude compared with D1 magnitude

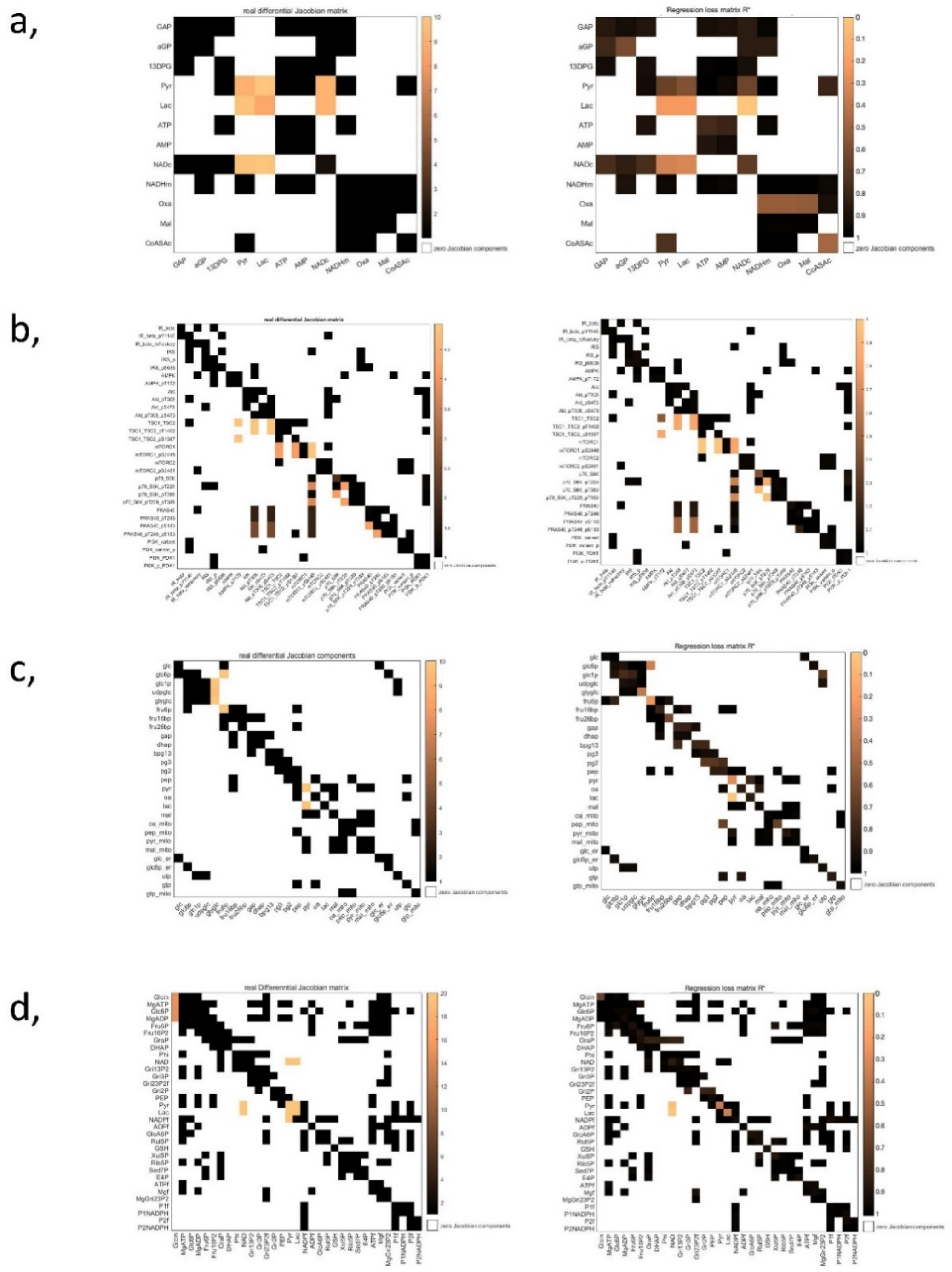

Supplementary Figure 3. The regression loss Jacobian algorithm evaluation assuming a diagonal fluctuation matrix D with $\varepsilon_D = 0.4$. For each test, all reactions are perturbed using a 0.3 magnitude fluctuation compared the metabolites fluctuation.

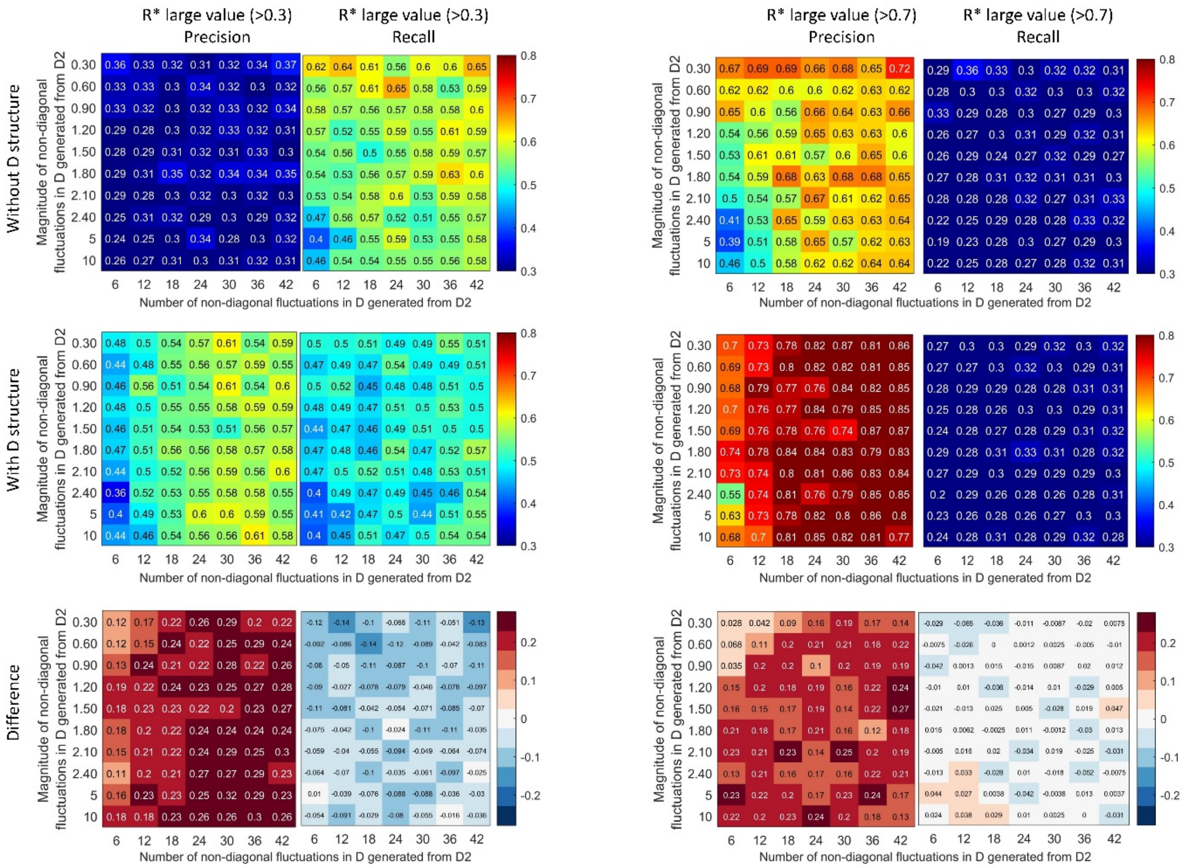

Supplementary Figure 4. Precision and Recall of the large values in R^{*} based on different thresholds (left: 0.3, right: 0.7). Color code refers to Figure 5A.

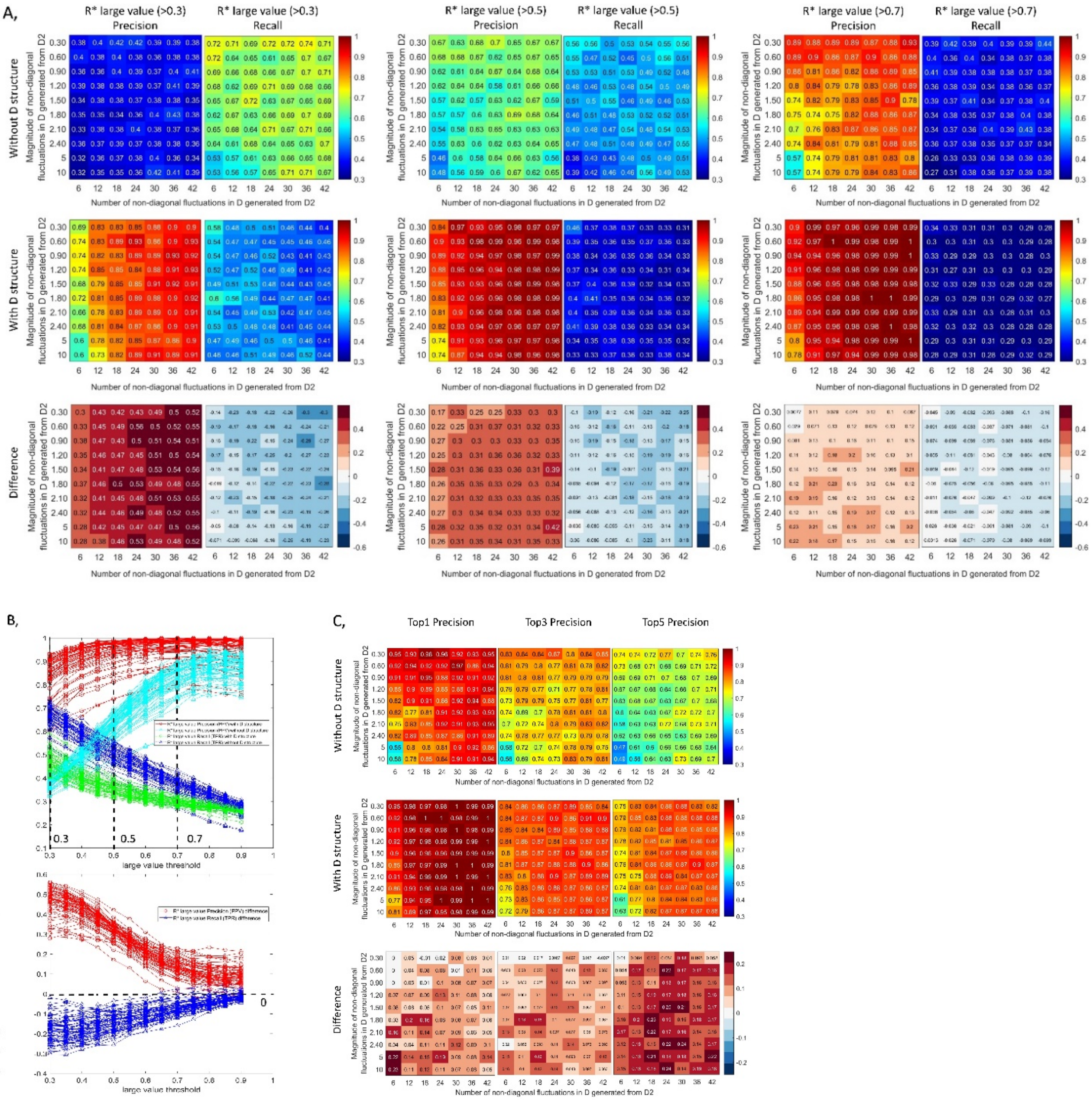

Supplementary Figure 5. Inverse differential Jacobian algorithm with/without D structure evaluation using various number and magnitude of off-diagonal fluctuations. The evaluation is conducted using the first model in method section with 200 repeats and $\varepsilon_D = 0.2$. A, Precision and Recall of the large values (above 0.5) in $\boldsymbol{R}^*$ over 200 repeats with/without D structure information; B, the line plots of Precision and Recall of the large values in $\boldsymbol{R}^*$ with/without D structure information based on different large value thresholds (0.3-0.9), where the snapshot of value 0.5 refers to A; C, the accuracy of the top 1, top 3 and top 5 large values in $\boldsymbol{R}^*$ over 200 repeats with/without D structure information.

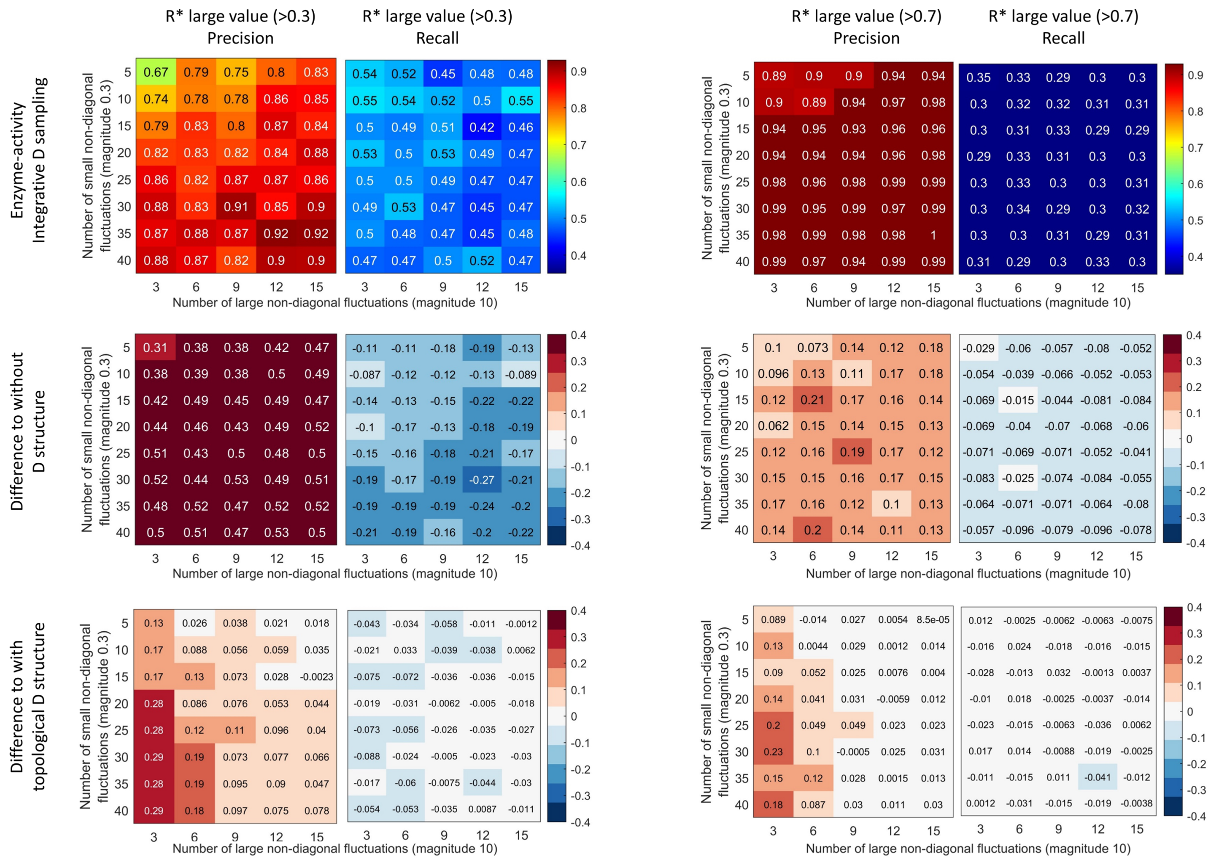

Supplementary Figure 6. Precision and Recall of the large values in R^* based on different thresholds (left: 0.3, right: 0.7). Color code refers to Figure 6A.

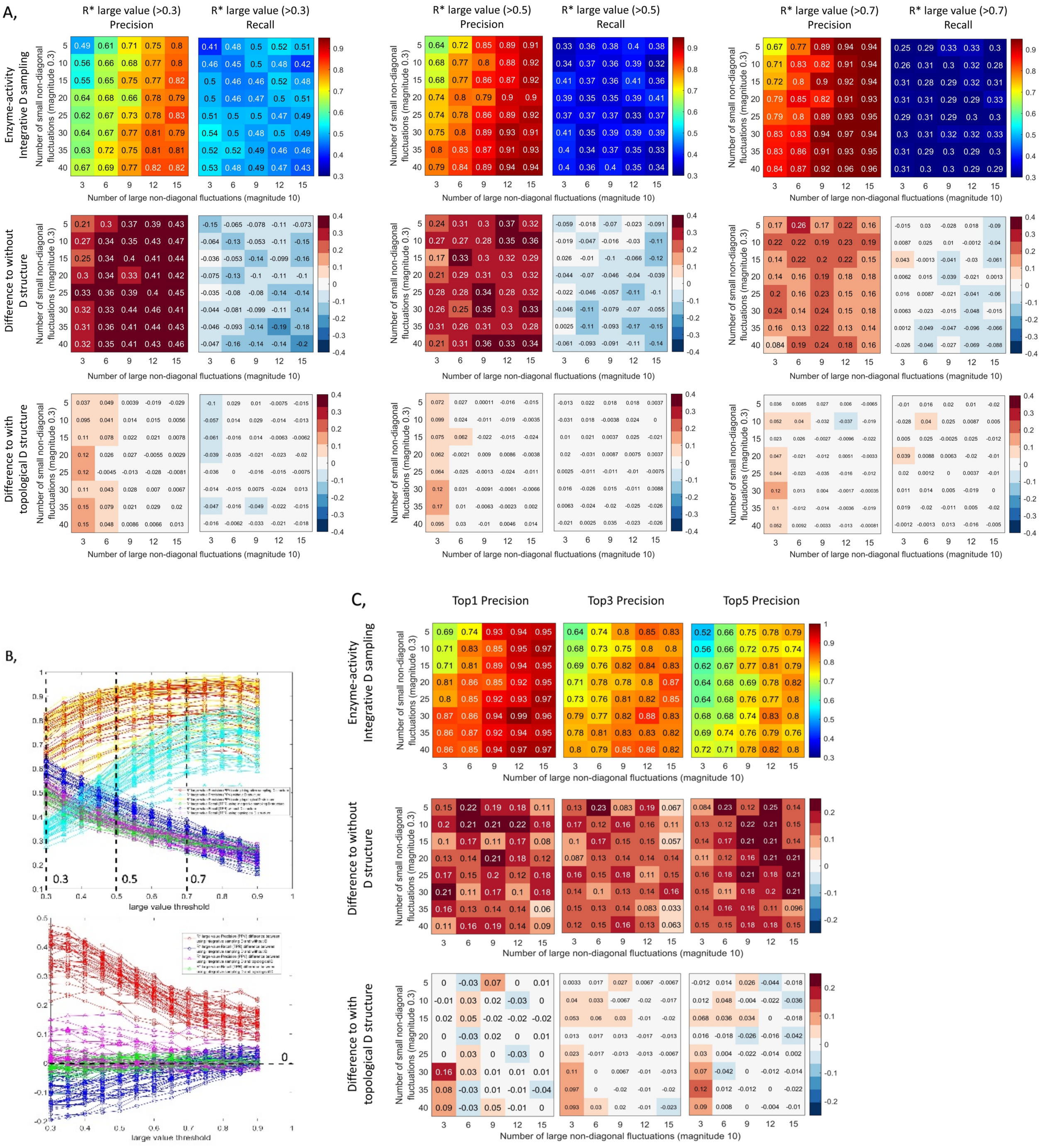

Supplementary Figure 7. Inverse differential Jacobian algorithm evaluation using various number of large (magnitude: 10) and small (magnitude: 0.3) non-diagonal fluctuations using three strategies: integrative D sampling, topological D and without D structure. The evaluation is conducted using the first model in method section with 200 repeats and $\varepsilon_D = 0.2$. A, Precision and Recall of the large values (above 0.5) in $\boldsymbol{R}^*$ over 200 repeats with/without D structure information; B, the line plots of Precision and Recall of the large values in $\boldsymbol{R}^*$ with/without D structure information based on different large value

thresholds (0.3-0.9), where the snapshot of value 0.5 refers to A; C, the accuracy of the top 1, top 3 and top 5 large values in $\boldsymbol{R}^{*}$ over 200 repeats with/without D structure information.

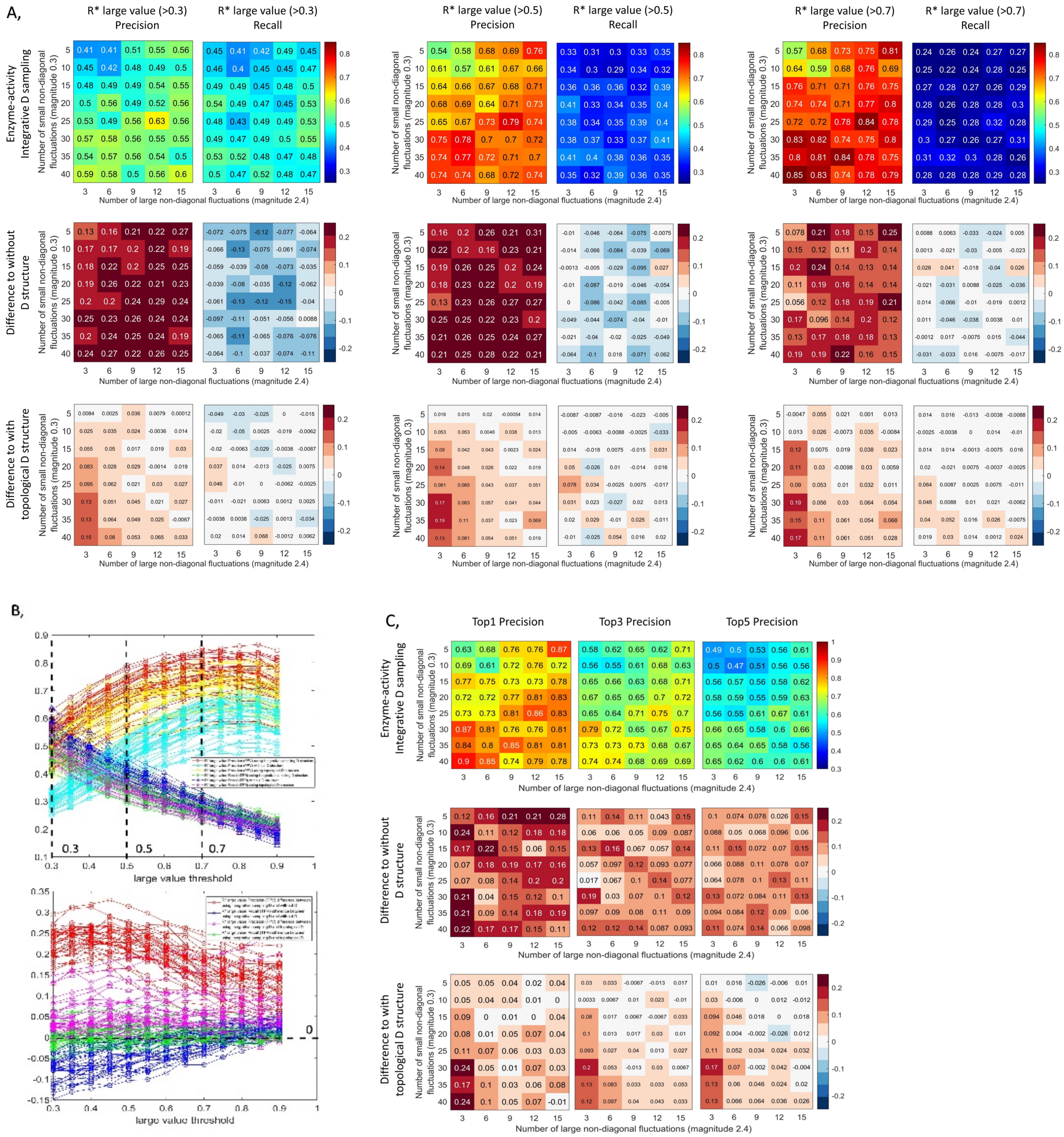

Supplementary Figure 8. Inverse differential Jacobian algorithm evaluation using various number of large (magnitude: 2.4) and small (magnitude: 0.3) non-diagonal fluctuations using three strategies: integrative D sampling, topological D and without D structure. The evaluation is conducted using the first model in method section with 200 repeats and $\varepsilon_D = 0.4$. A, Precision and Recall of the large values (above 0.5) in $\boldsymbol{R}^*$ over 200 repeats with/without D structure information; B, the line plots of Precision and Recall of the large values in $\boldsymbol{R}^*$ with/without D structure information based on different large value

thresholds (0.3-0.9), where the snapshot of value 0.5 refers to A; C, the accuracy of the top 1, top 3 and top 5 large values in $\boldsymbol{R}^*$ over 200 repeats with/without D structure information.

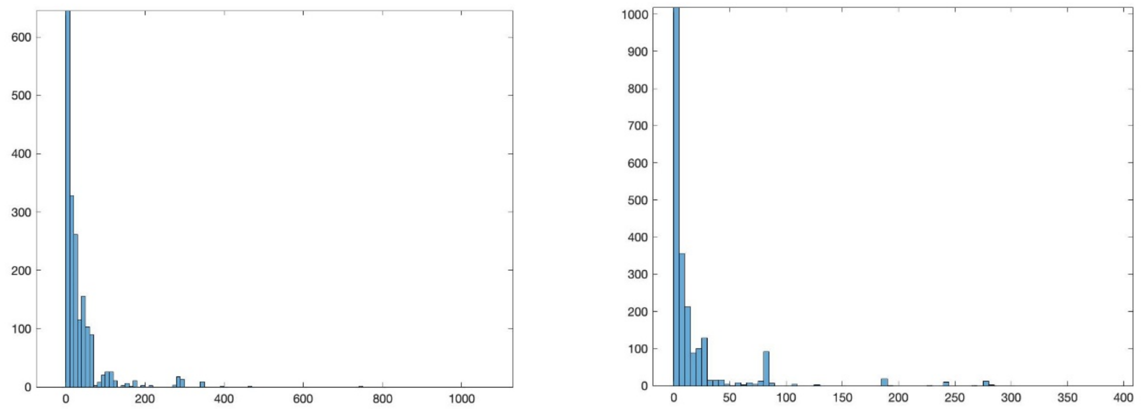

Supplementary Figure 9. The histogram plots of the activities variances of the two cell lines enzymes.

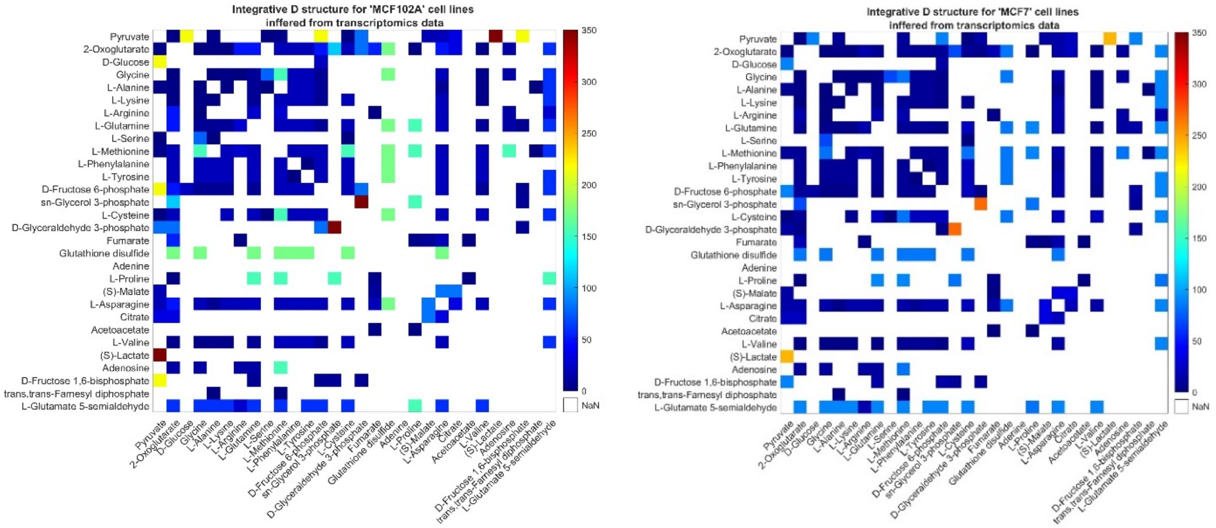

Supplementary Figure 10. The inferred fluctuation matrix structure for the two different cell lines (left: $\mathrm{D_h}$ for MCF 102A and right: $\mathrm{D_d}$ for MCF 7).

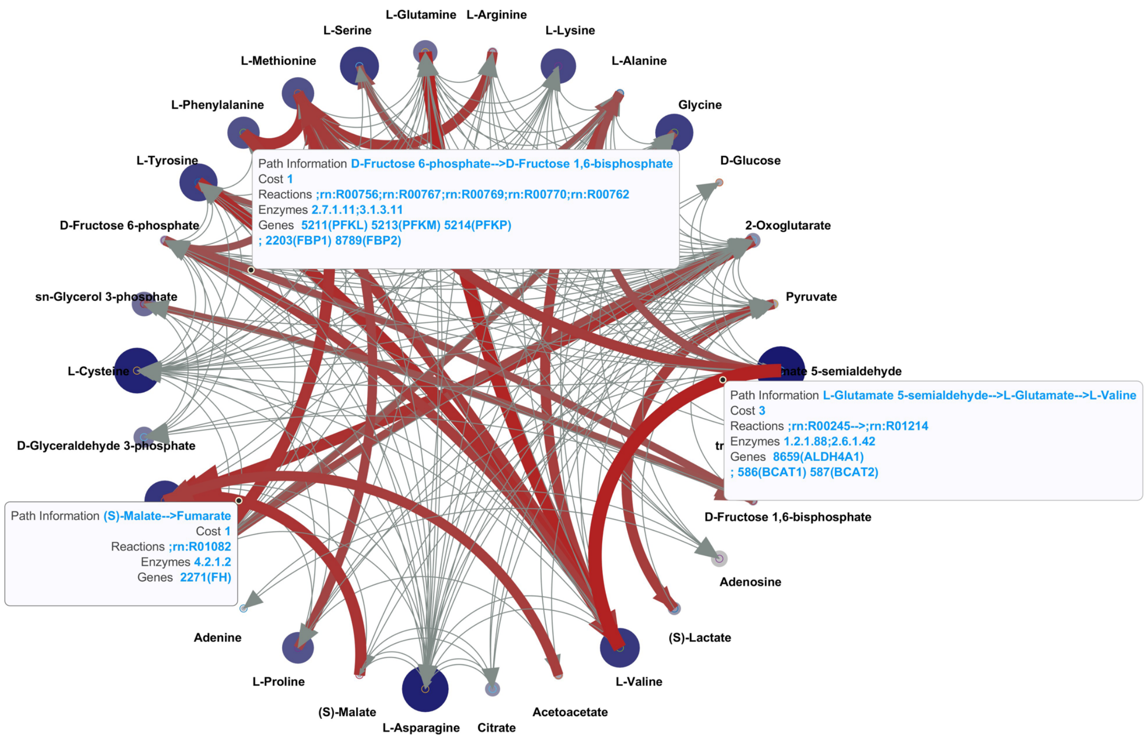

L-Glutamine
L-Arginine
L-Serine
L-Lysine
L-Methionine
L-Alanine
L-Phenylalanine
Glycine
L-Tyrosine
D-Glucose
Path Information D-Fructose 6-phosphate-->D-Fructose 1,6-bisphosphate
Cost 1
Reactions ;rn:R00756;rn:R00767;rn:R00769;rn:R00770;rn:R00762
Enzymes 2.7.1.11;3.1.3.11
Genes 5211(PFKL) 5213(PFKM) 5214(PFKP)
; 2203(FBP1) 8789(FBP2)
D-Fructose 6-phosphate
2-Oxoglutarate
sn-Glycerol 3-phosphate
Pyruvate
L-Cysteine
Path Information L-Glutamate 5-semialdehyde-->L-Glutamate-->L-Valine
Cost 3
Reactions ;rn:R00245-->;rn:R01214
Enzymes 1.2.1.88;2.6.1.42
Genes 8659(ALDH4A1)
; 586(BCAT1) 587(BCAT2)
D-Glyceraldehyde 3-phosphate
Path Information (S)-Malate-->Fumarate
Cost 1
Reactions ;rn:R01082
Enzymes 4.2.1.2
Genes 2271(FH)
D-Fructose 1,6-bisphosphate
Adenosine
Adenine
(S)-Lactate
L-Proline
L-Valine
(S)-Malate
Acetoacetate
L-Asparagine
Citrate

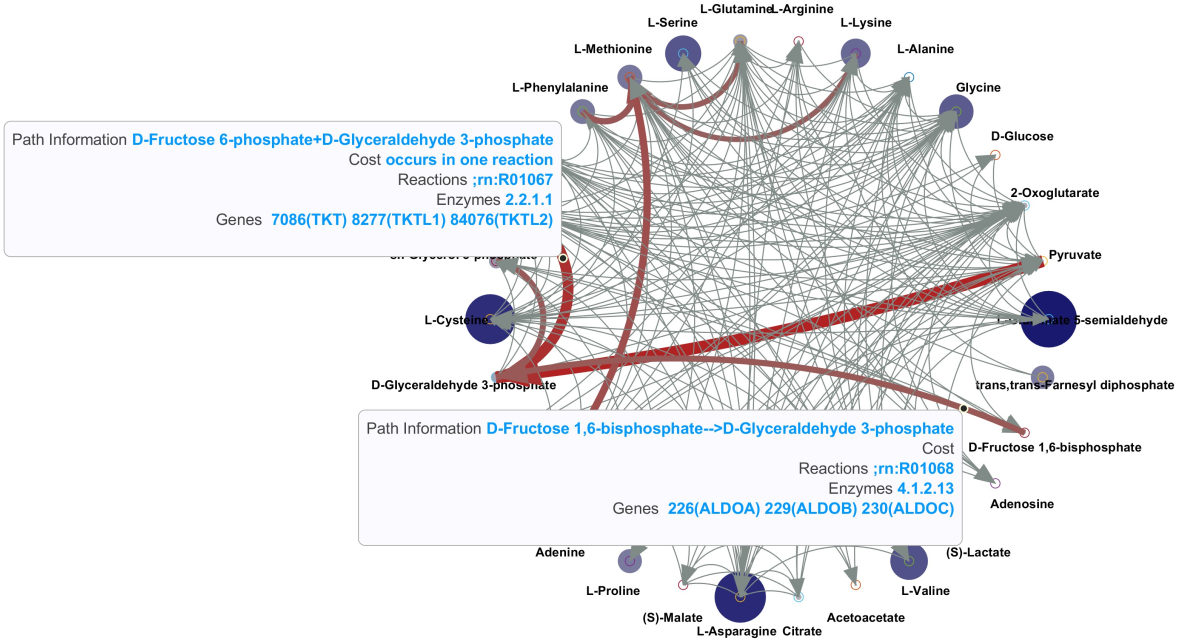

L-Glutamine
L-Arginine
L-Serine
L-Lysine
L-Methionine
L-Alanine
L-Phenylalanine
Glycine
Path Information D-Fructose 6-phosphate+D-Glyceraldehyde 3-phosphate
Cost occurs in one reaction
Reactions ;rn:R01067
Enzymes 2.2.1.1
Genes 7086(TKT) 8277(TKTL1) 84076(TKTL2)
D-Glucose
2-Oxoglutarate
Pyruvate
L-Cysteine
D-Glyceraldehyde 3-phosphate
trans,trans-Farnesyl diphosphate
Path Information D-Fructose 1,6-bisphosphate-->D-Glyceraldehyde 3-phosphate
Cost
Reactions ;rn:R01068
Enzymes 4.1.2.13
Genes 226(ALDOA) 229(ALDOB) 230(ALDOC)
D-Fructose 1,6-bisphosphate
Adenosine
Adenine
(S)-Lactate
L-Proline
L-Valine
(S)-Malate
Acetoacetate
L-Asparagine
Citrate

Supplementary Figure 11. The interactive plots of breast cancer inverse Jacobian analysis. Top plot uses a diagonal D assumption and bottom plot uses enzyme-activity integrative D sampling. The circular plot can be interactively checked by clicking the interactions (lines) through the matlab figure format result available in Supplementary material S2.

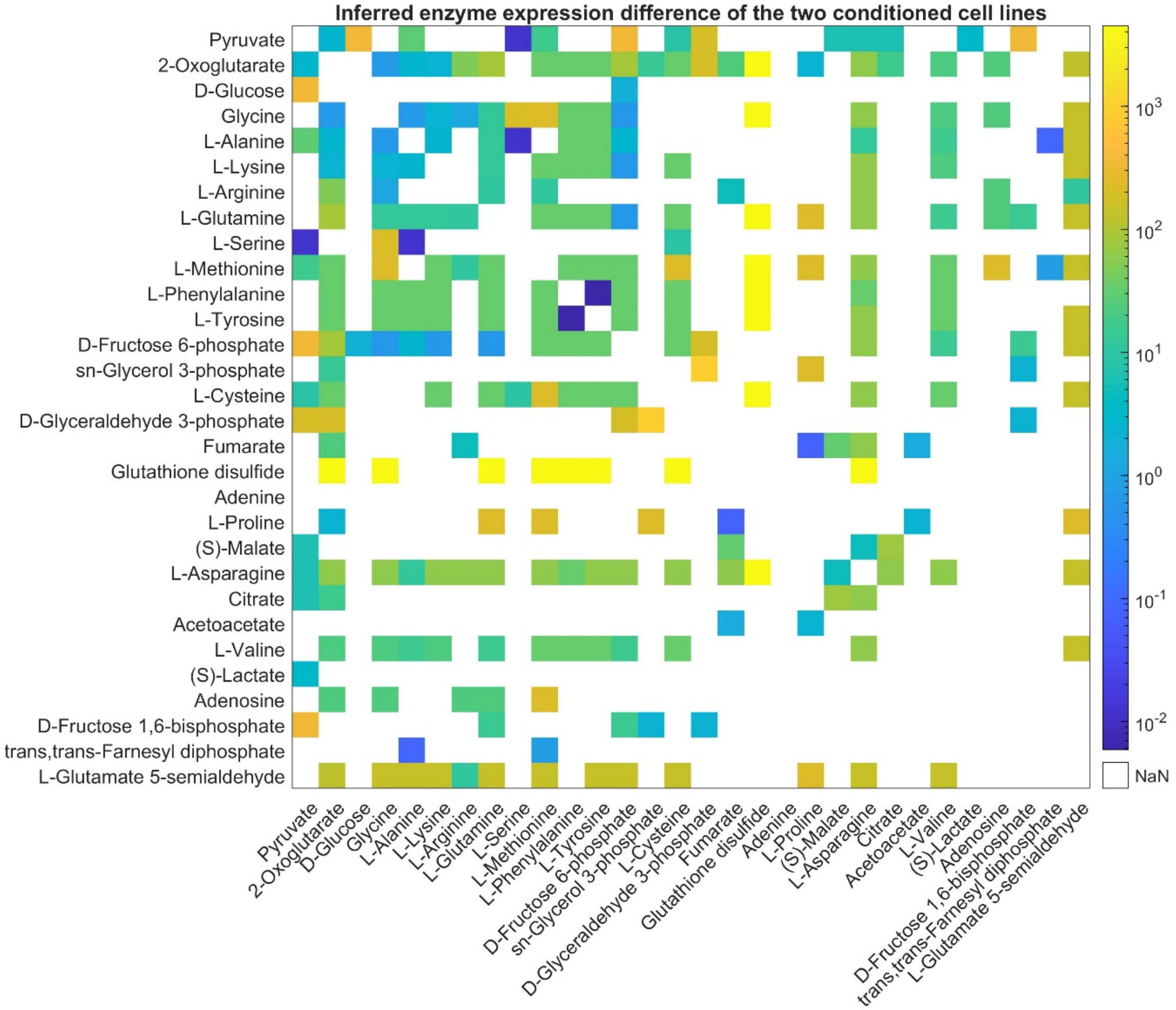

Supplementary Figure 12. The inferred enzyme expression difference of the two conditioned cell lines.
